# Mechanism of Electrocatalytic H_2_ Evolution, Carbonyl Hydrogenation, and Carbon–Carbon Coupling on Cu

**DOI:** 10.1021/jacs.4c01911

**Published:** 2024-05-13

**Authors:** Hongwen Chen, Jayendran Iyer, Yue Liu, Simon Krebs, Fuli Deng, Andreas Jentys, Debra J. Searles, M. Ali Haider, Rachit Khare, Johannes A. Lercher

**Affiliations:** † Department of Chemistry and Catalysis Research Center, Technical University of Munich, Garching 85748, Germany; ‡ Renewable Energy and Chemicals Laboratory, Department of Chemical Engineering, Indian Institute of Technology Delhi, New Delhi 110016, India; § Australian Institute for Bioengineering and Nanotechnology, The University of Queensland, Brisbane 4072, QLD, Australia; ∥ Shanghai Key Laboratory of Green Chemistry and Chemical Processes, School of Chemistry and Molecular Engineering, East China Normal University, Shanghai 200062, China; ⊥ School of Chemistry and Molecular Biosciences, The University of Queensland, Brisbane 4072, QLD, Australia; # ARC Centre of Excellence for Green Electrochemical Transformation of Carbon Dioxide, The University of Queensland, Brisbane 4072, QLD, Australia; ∇ Indian Institute of Technology Delhi−Abu Dhabi, Khalifa City B, Abu Dhabi, United Arab Emirates; ○ Institute for Integrated Catalysis, Pacific Northwest National Laboratory, Richland 99352, Washington, United States

## Abstract

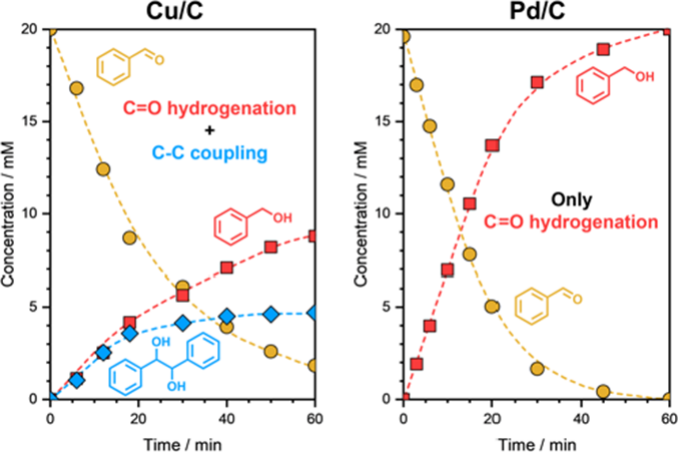

Aqueous-phase electrocatalytic hydrogenation of benzaldehyde on Cu leads not only to benzyl alcohol (the carbonyl hydrogenation product), but Cu also catalyzes carbon–carbon coupling to hydrobenzoin. In the absence of an organic substrate, H_2_ evolution proceeds via the Volmer–Tafel mechanism on Cu/C, with the Tafel step being rate-determining. In the presence of benzaldehyde, the catalyst surface is primarily covered with the organic substrate, while H* coverage is low. Mechanistically, the first H addition to the carbonyl O of an adsorbed benzaldehyde molecule leads to a surface-bound hydroxy intermediate. The hydroxy intermediate then undergoes a second and rate-determining H addition to its α-C to form benzyl alcohol. The H additions occur predominantly via the proton-coupled electron transfer mechanism. In a parallel reaction, the radical α-C of the hydroxy intermediate attacks the electrophilic carbonyl C of a physisorbed benzaldehyde molecule to form the C–C bond, which is rate-determining. The C–C coupling is accompanied by the protonation of the formed alkoxy radical intermediate, coupled with electron transfer from the surface of Cu, to form hydrobenzoin.

## Introduction

Upgrading lignocellulosic biomass to value-added chemicals is an emergent technology that will contribute to replacing crude oil as a carbon source for energy carriers and fine chemicals.^1−4^ Upgrading biomass typically involves the reduction of biomass-derived oxygenated compounds^5,6^ (to increase their stability) and C–C coupling^7,8^ (to increase the molecular weight). Aqueous-phase electrochemical reduction, i.e., hydrogenation (ECH) of biomass-derived oxygenates, such as alcohols, phenols, aldehydes, ketones, and carboxylic acids, is one of the promising strategies.^9−11^ ECH has been shown to successfully reduce the aromatic rings in aromatics,^12,13^ carbonyl groups in aldehydes and ketones,^14−16^ as well as C=C double bonds in unsaturated carboxylic acids.^17,18^ ECH, in addition to reduction, has also been shown to catalyze C–C bond formation in benzaldehyde and furfural derivates.^19,20^

The upgrading of biomass to liquid biofuels via thermocatalytic hydrogenation (TCH) requires elevated H_2_ pressure and high temperatures.^21,22^ ECH, on the other hand, generates hydrogen in situ from an aqueous electrolyte under an external electric potential, which typically leads to high equilibrium pressures. Rather than forming H_2_, the formed surface hydrogen atoms (H*) can also react with (the biomass-derived) organic substrates under mild reaction conditions. ECH, therefore, has several advantages, including lower operating temperatures, no requirement for external H_2_ supply, and easy integration with renewable power harvesting.^23,24^

The carbonyl functional groups that are abundantly present in bio-oils, predominantly as aromatic aldehydes and ketones, are prone to polymerization. The reduction of these carbonyl groups is therefore required to increase the stability of bio-oils. Benzaldehyde (BZ), the simplest aromatic aldehyde, is an ideal model compound to investigate low-temperature electroreduction of carbonyl groups present in the bio-oils. Furthermore, BZ and furfural derivatives, following electroreductive C–C coupling, have been suggested as potential sources of high-quality fuel and value-added chemicals.^25−27^

**Scheme 1:**
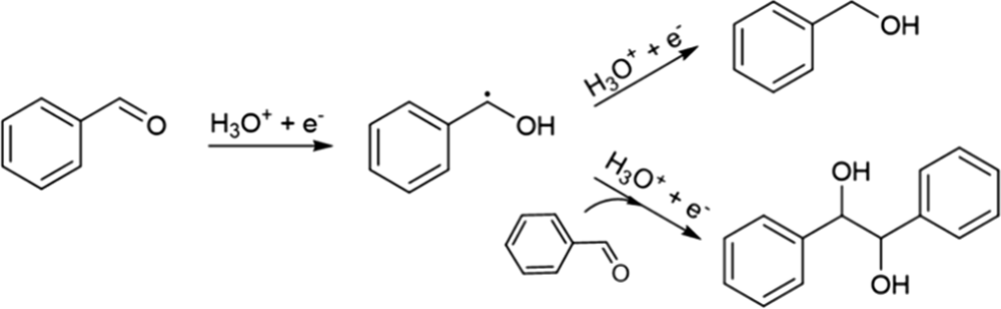
Hydrogenation of BZ to BA and HB

BZ hydrogenation, both thermochemical and electrochemical, has been investigated in detail on noble metals (e.g., Pt, Pd, Ru, and Rh) as well as base metals (e.g., Cu, Ni, and Pb).^19,28−32^Scheme 1 shows the two possible products resulting from H addition and C–C coupling, viz., benzyl alcohol (BA) and hydrobenzoin (HB). Although several studies have investigated BA formation in detail, limited effort has been devoted to understanding electrochemical C–C coupling to produce HB. A primary reason for this is that HB has been observed as a product only on a few catalysts.^20,33,34^

ECH of BZ to BA (and to HB when coupled with C–C bond formation) requires two successive H addition steps. The H addition has been proposed to proceed via two distinct mechanisms: (i) direct hydrogenation of the adsorbed organic substrate by a surface hydride species (typically proceeding via Langmuir–Hinshelwood (LH) kinetics, often referred to as the LH mechanism)^30^ or (ii) proton-coupled electron-transfer (PCET) mechanism.^28,35^ Koh et al. proposed the PCET mechanism for BZ hydrogenation on Pd.^35^ Singh et al., on the other hand, investigated aqueous-phase hydrogenation of BZ on Pt group metals and proposed the LH mechanism for H addition.^29^

The effects of solvents and coreactants on BZ hydrogenation have also been investigated. For example, Sanyal et al. showed that the presence of polar coadsorbates such as phenol enhances the rate of BZ ECH on carbon-supported metals via hydrogen bonding.^36^ More recently, Cheng et al. investigated the role of solvents in the catalytic hydrogenation of BZ and showed that solvents affect the binding strength of adsorbed H, resulting in different hydrogenation rates.^37^ Overall, the kinetics and mechanism of BZ ECH to BA have been extensively investigated, both experimentally and computationally.^30,38−40^

In comparison to BA formation, the reaction path for the reductive electrocatalytic conversion of BZ to HB was only speculatively addressed. It has been proposed that C–C coupling occurs primarily via the LH mechanism involving the reaction of two surface ketyl radicals.^19,20^ The ability of a catalyst to form and stabilize the ketyl intermediate has been proposed as a key descriptor of its ability to promote C–C coupling.^33^ Interestingly, evidence for ketyl radicals was observed on Cu, which promotes C–C coupling, but not on Pd or Pt.^33^

With the goal of understanding these mechanistic pathways, we report here a detailed kinetic and mechanistic study of ECH of BZ to both BA and HB on Cu/C. Using isotope labeling studies, we show that the rate-determining step for BA formation is the second H addition, while that for HB formation is the C–C coupling. Combining experimental evidence with molecular simulations using periodic density functional theory (DFT), we postulate the possible reaction pathways for HB, BA, and H_2_ formation during BZ ECH on Cu/C.

## Results and Discussion

Cu/C catalyst (∼5 wt % metal loading) was synthesized via the incipient-wetness impregnation method using copper(II) acetate as the Cu source and Vulcan carbon black as the support. The specific surface area of the catalyst was estimated to be ∼223 m^2^·g_cat_^–1^ from N_2_ adsorption–desorption measurements (Supplementary Figure S1). The formation of metallic Cu nanoparticles in the Cu^0^ oxidation state was confirmed by X-ray absorption near-edge structure (XANES) measurements (Supplementary Figure S2a). Furthermore, the extended X-ray absorption fine structure (EXAFS) analysis indicated a Cu–Cu coordination number of ∼10 (Supplementary Figure S2b and Table S1), suggesting the formation of large Cu nanoparticles. The formation of large nanoparticles was also confirmed by transmission electron microscopy (TEM) images of the synthesized catalyst (Supplementary Figure S3). Lastly, X-ray diffraction (XRD) measurements of Cu/C indicated Cu(111) to be the most abundant facet in the formed Cu nanoparticles (Supplementary Figure S4).

### H_2_ Evolution Reaction on Cu in the Absence of BZ

**Figure 1:**
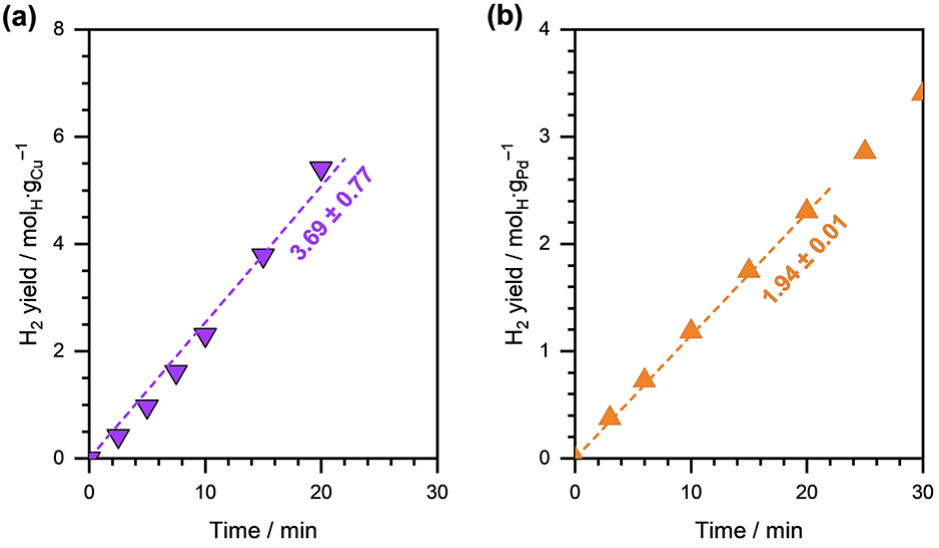
H_2_ evolution as a function of reaction time during HER on (a) Cu/C and (b) Pd/C. Reaction conditions: η = −0.5 V vs RHE on Cu/C and η = −0.2 V vs RHE on Pd/C, 1.5 M acetate buffer solution (pH ∼ 4.6), room temperature, ambient pressure. The dashed lines are linear fits, and the reported numbers are initial HER rates in mmol_H_·g_metal_^–1^·s^–1^.

We first investigated the H_2_ evolution reaction (HER) on Cu/C in the absence of an organic substrate. Figure 1 shows the H_2_ yield (in mol H consumed per gram metal) as a function of reaction time at an applied external potential (η) of −0.5 V versus RHE on Cu/C. The H_2_ yield during HER on Pd/C at η = −0.2 V versus RHE is also shown for comparison. The HER rate on Cu/C was estimated to be ∼3.7 mmol_H_·g_Cu_^–1^·s^–1^, while the HER rate on Pd/C was ∼1.9 mmol_H_·g_Pd_^–1^·s^–1^. A significantly higher overpotential required to achieve similar HER rates on Cu/C suggests a weaker hydrogenation ability of Cu, compared to Pd.

### Mechanism of H_2_ Evolution on Cu in the Absence of BZ

**Scheme 2:**
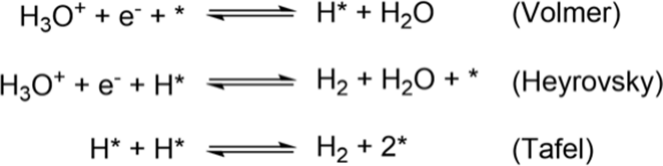
Volmer, Heyrovsky, and Tafel Steps for HER

The H_2_ evolution on metal electrodes has been described in terms of the Volmer–Heyrovsky–Tafel mechanism (illustrated in Scheme 2).^41^ The Volmer step is a PCET step that involves the adsorption of a solvated proton (H_3_O^+^) on the surface of the electrode, coupled with simultaneous electron transfer from the surface, to form H*. The Heyrovsky step is an Eley–Rideal (ER)-type PCET step wherein the surface H* reacts with a solvated proton and an electron to form H_2_. The Tafel step, on the other hand, is an LH-type surface recombination of two H* to form H_2_.

Both Volmer and Heyrovsky steps follow the PCET mechanism; therefore, their rates can be expressed as
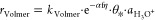
1
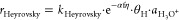
2where *k*_Volmer_ and *k*_Heyrovsky_ are the respective kinetic rate constants, α is the electron-transfer coefficient, *f* denotes *F*/*RT* (where *F*, *R*, and *T* denote the Faraday constant, the universal gas constant, and the temperature, respectively), θ_*_ and θ_H_ are the surface coverage of empty sites (*) and H*, respectively, and *a*_H_3_O^+^_ is the activity of hydronium ions (equal to their concentration under these conditions). In contrast, the rate of the LH-type Tafel step is expressed as
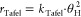
3where *k*_Tafel_ is the kinetic rate constant of the Tafel step, and θ_H_ is the surface coverage of H*.

To determine the kinetically rate-determining step (rds) for H_2_ evolution on Cu, we estimated the Tafel slope of HER on Cu/C in the pure electrolyte solution. Theoretically (at *T* = 298 K and α = 0.5), the Tafel slope is equal to ∼120 mV·dec^–1^ if the Volmer step is the rate-determining step.^41^ On the other hand, if the rate-determining step is the Heyrovsky step or the Tafel step, the Tafel slope is equal to ∼40 or ∼30 mV dec^–1^, respectively.^41,42^ Furthermore, at high H* coverages (i.e., θ_H_ ≈ 1), theoretically, the Tafel slope becomes equal to ∼120 mV·dec^–1^ in the case of the Heyrovsky step being rate-determining or equal to ∞ if the Tafel step is rate-determining.^41,42^

**Figure 2:**
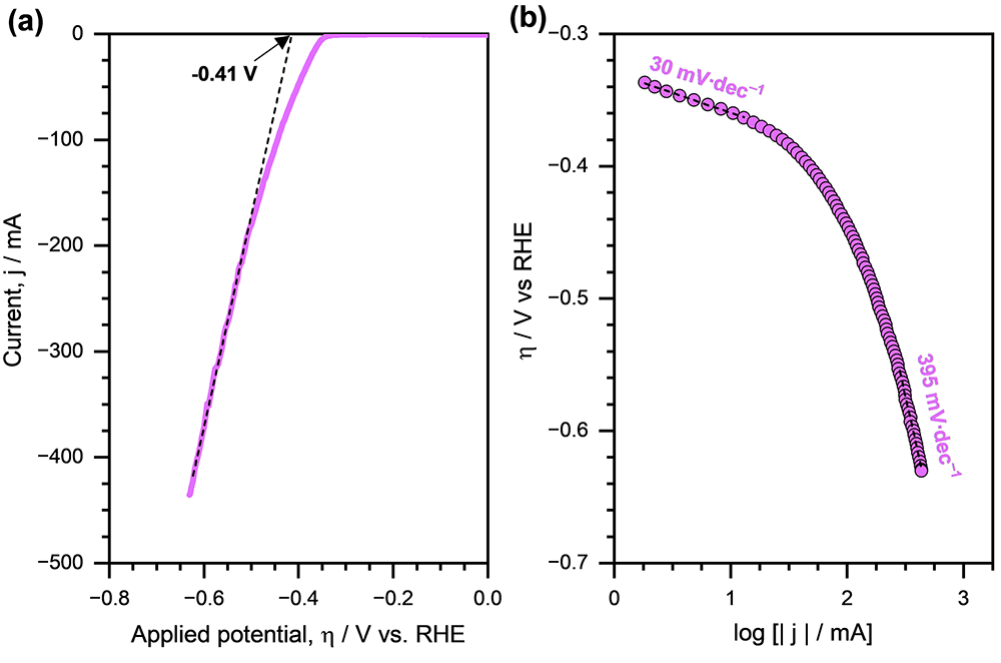
(a) Linear sweep voltammetry curve (scan rate = 1 mV·s^–1^) of HER in pure electrolyte on Cu/C. (b) Tafel curve of HER in pure electrolyte on Cu/C. The dashed lines are linear fits, and the reported numbers are Tafel slopes. Reaction conditions: 1.5 M acetate buffer solution (pH ∼ 4.6), room temperature, ambient pressure.

Figure 2a shows the linear sweep voltammetry (LSV) curve of HER on Cu/C. The corresponding Tafel curve is presented in Figure 2b. Based on the LSV curve, we estimated the onset potential of HER on Cu/C to be approximately equal to −0.41 V versus RHE. Additionally, we noted that at low overpotentials (i.e., η > −0.4 V vs RHE), the Tafel slope was equal to ∼30 mV dec^–1^, clearly suggesting that the Tafel step is the rate-determining step for H_2_ evolution. In other words, the HER on Cu/C (in the absence of organic substrate) proceeds via the Volmer–Tafel (rds) pathways under the applied reaction conditions. We also note that the value of Tafel slope increased with the increasing overpotential, reaching ∼395 mV·dec^–1^ at η = −0.6 V versus RHE. The value of the Tafel slope was equal to ∼250 mV·dec^–1^ at η = −0.5 V versus RHE. These higher values of the Tafel slope indicate a high surface coverage of H* at higher overpotentials.^41,42^

**Figure 3:**
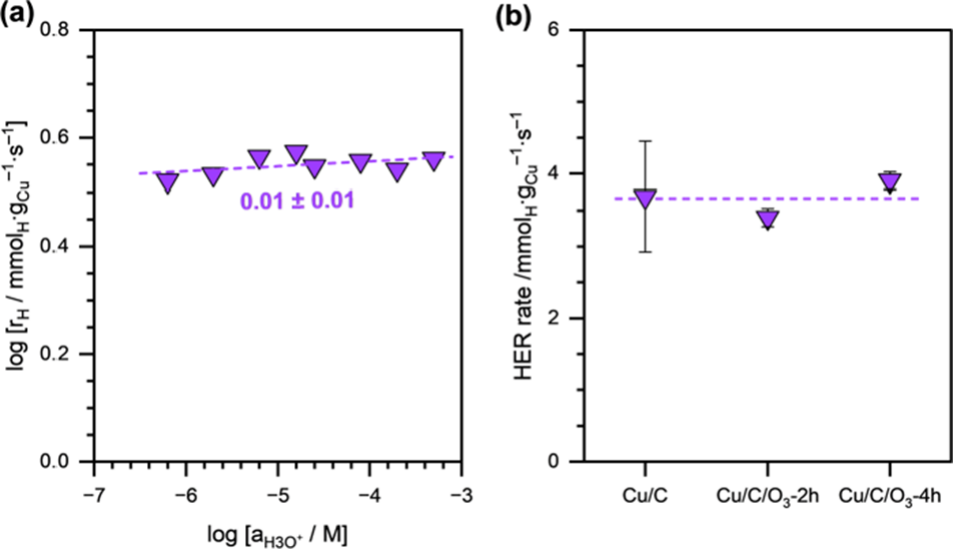
(a) HER rates as a function of *a*_H_3_O^+^_ (pH 3.2–6.3) on Cu/C. The dashed line is a linear fit, and the reported number is the calculated reaction order. (b) HER rates in a pure electrolyte solution on Cu/C, Cu/C/O_3_-2h, and Cu/C/O_3_-4h catalysts at pH ∼ 4.6. The dashed line is a guide to the eye. Reaction conditions: η = −0.5 V vs RHE, 1.5 M acetate buffer solution (pH ∼ 4.6), room temperature, ambient pressure.

We also investigated the effect of hydronium ion concentration (*a*_H_3_O^+^_) on HER by varying the pH of the electrolyte solution (shown in Figure 3a). We can clearly see that the HER rate showed almost no dependence on *a*_H_3_O^+^_, i.e., the reaction order was approximately zero. Furthermore, we also performed HER on Cu/C catalysts with an acid-functionalized support. Acid-functionalization of the support via O_3_ treatment has been shown to increase *a*_H_3_O^+^_ near the surface of the electrode, thus enhancing the rates of elementary steps involving PCET.^35^Figure 3b shows the effect of O_3_ treatment of the carbon support on the HER rates. Again, we can clearly see that the HER rates remained unchanged after O_3_ treatment.

**Scheme 3:**
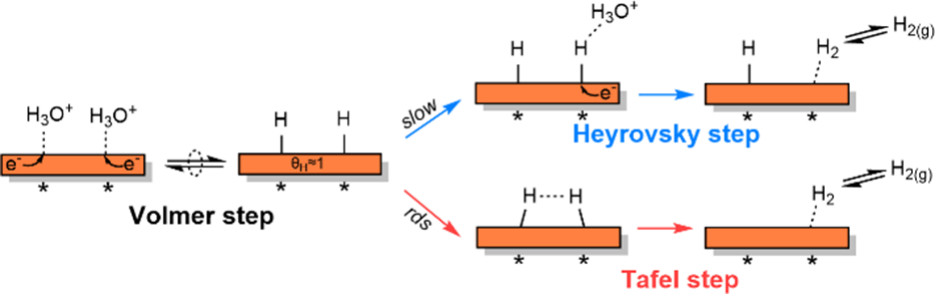
Reaction Mechanism for H_2_ Evolution on Cu/C in the Absence of BZ

From eqs 1 and 2, it can be deduced that *r*_Volmer_ and *r*_Heyrovsky_ are dependent on η and *a*_H_3_O^+^_. On the other hand, *r*_Tafel_ is independent of η or *a*_H_3_O^+^_ (eq 3) at high H* coverages (i.e., θ_H_ ≈ 1). It must however be noted that at low coverages of H* (i.e., when θ_H_ ≪ 1), θ_H_ itself varies with η and a_H_3_O^+^_ (due to its dependence on the reversible rate of the Volmer step). Therefore, *r*_Tafel_ indirectly depends on η and *a*_H_3_O^+^_ when θ_H_ ≪ 1. The zero-order dependence of HER on *a*_H_3_O^+^_ (Figure 3a), therefore, suggests high H* on the surface of Cu (i.e., θ_H_ ≈ 1). The Volmer step, therefore, can be assumed to be equilibrated under these reaction conditions. Furthermore, the invariance of HER rates with *a*_H_3_O^+^_ clearly suggests that the kinetically relevant step for H_2_ evolution on Cu/C is not a PCET step. As both Volmer and Heyrovsky steps follow the PCET mechanism, we conclude that the rate-determining step for H_2_ evolution on Cu/C, in the absence of an organic substrate, is the Tafel step, and HER on Cu follows the Volmer–Tafel(rds) pathway. Scheme 3 illustrates the postulated mechanism for H_2_ evolution on Cu/C in the absence of an organic substrate.

We must note here that, based on the obtained Tafel slopes, the rate-determining step for HER on Cu-based electrocatalysts has also been proposed to be the Volmer step or the Heyrovsky step under different reaction conditions. For example, Sharifi-Asl et al. suggested the Volmer step to be the rate-determining step (Tafel slope = 87–120 mV·dec^–1^) for HER on pure Cu electrodes.^43^ The higher Tafel slope in their case is likely due to low *a*_H_3_O^+^_ (pH = 5.7–9.2) employed in their studies. Xue et al., on the other hand, concluded that the HER on Cu@graphdiyne core–shell electrocatalysts proceeds via the Volmer–Heyrovsky(rds) mechanism (Tafel slope ∼69 mV·dec^–1^).^44^ We speculate that the higher Tafel slope in this case could be due to the higher scan rate (5 mV·s^–1^) employed during the LSV measurements. The scan rate has been shown to affect the Tafel slopes in the potentiodynamic LSV measurements.^45^ Interestingly, it has been proposed that the HER proceeds via the Volmer–Tafel(rds) mechanism on Cu-based metal organic frameworks.^46,47^ Based on the kinetic investigations and LSV measurements, we conclude that the Tafel step is the rate-determining step for HER on Cu/C and that the HER proceeds via the Volmer–Tafel(rds) pathway on Cu/C.

### ECH of BZ on Cu

**Figure 4:**
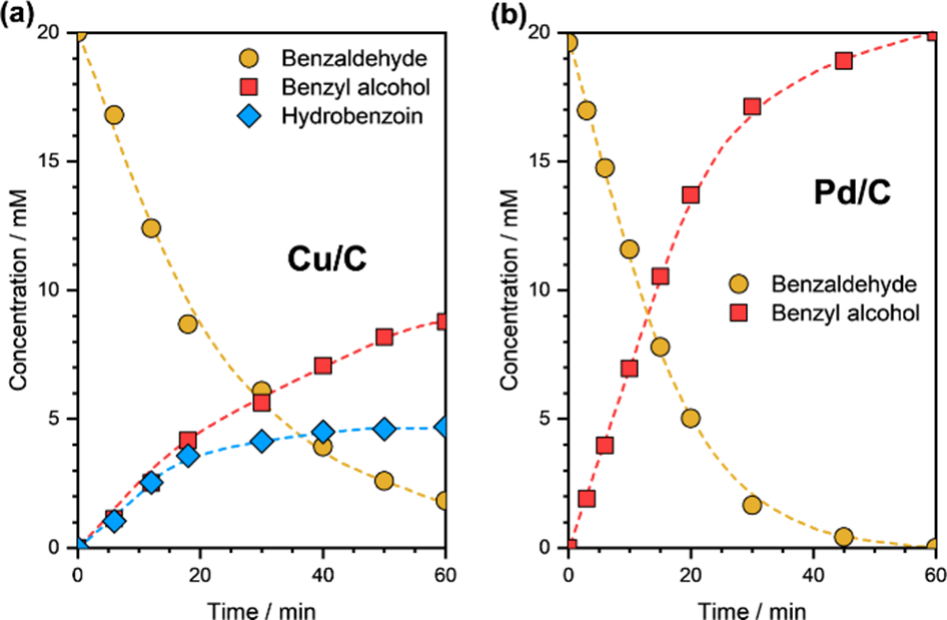
Concentration profiles of reactants and products during BZ ECH on (a) Cu/C and (b) Pd/C. Reaction conditions: 20 mM BZ, η = −0.5 V vs RHE on Cu/C and η = −0.2 V vs RHE on Pd/C, 1.5 M acetate buffer solution (pH ∼ 4.6), room temperature, ambient pressure. The dashed lines are guides to the eye.

Let us now look at the aqueous-phase ECH of BZ on Cu/C. The LSV curve of BZ ECH on Cu/C is presented in Supplementary Figure S5. The onset potential of BZ ECH on Cu/C was estimated to be −0.4 V versus RHE. Figure 4 shows the concentration profiles of reactants and products during BZ ECH on Cu/C at η = −0.5 V versus RHE. The concentration profiles during BZ ECH on Pd/C (at η = −0.2 V vs RHE) are also shown. First of all, it is noteworthy that Cu, unlike Pd, in addition to C=O hydrogenation to BA, catalyzed C–C coupling to HB. No other byproducts were observed. Based on the yield versus conversion plots (Supplementary Figure S6), it can be established that both BA and HB are kinetically primary products. We must also mention here that the carbon support showed negligible activity toward BZ ECH under the same reaction conditions (Supplementary Figure S7).

**Figure 5:**
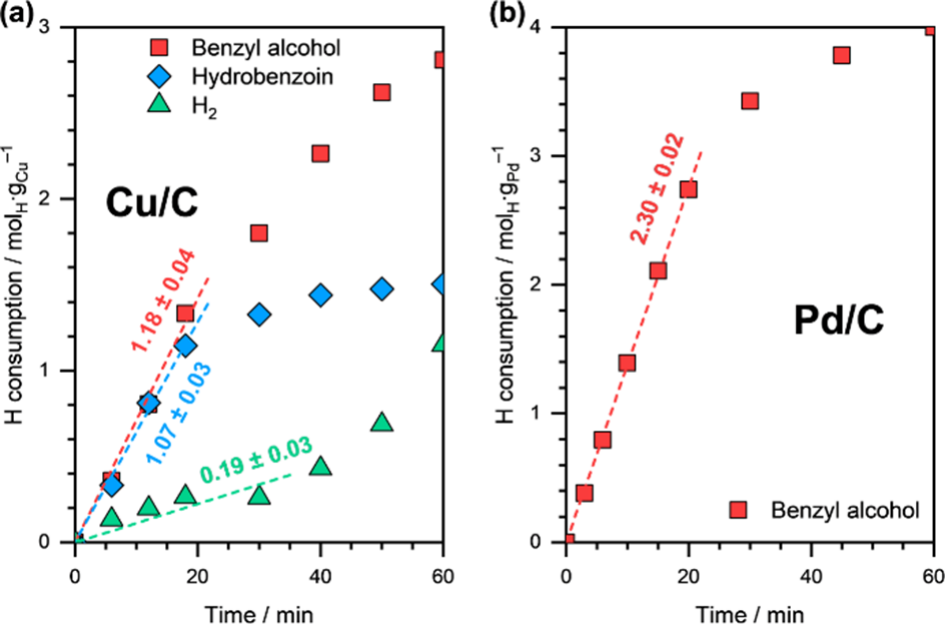
H consumption toward BA, HB, and H_2_ formation as a function of reaction time during BZ ECH on (a) Cu/C and (b) Pd/C. Reaction conditions: 20 mM BZ, η = −0.5 V vs RHE on Cu/C and η = −0.2 V vs RHE on Pd/C, 1.5 M acetate buffer solution (pH ∼ 4.6), room temperature, ambient pressure. The dashed lines are linear fits, and the reported numbers are the initial rates in mmol_H_·g_metal_^–1^·s^–1^.

Figure 5 shows the H consumption toward different products as a function of reaction time during BZ ECH on Cu/C and Pd/C. The total H consumption rate on Pd/C was estimated to be ∼2.3 mmol_H_·g_Pd_^–1^·s^–1^, and the overall Faradaic efficiency toward BZ conversion was almost 100%. Interestingly, the total H consumption rate was similar on Cu/C (∼2.4 mmol_H_·g_Cu_^–1^·s^–1^) albeit at a much higher overpotential, again indicating the weaker hydrogenation ability of Cu compared to Pd. However, similar rates of BA and HB formation on Cu (∼1.2 and ∼1.1 mmol_H_·g_Cu_^–1^·s^–1^, respectively) highlight its remarkable ability to catalyze both C=O hydrogenation and C–C coupling reactions. The overall Faradaic efficiency of Cu/C toward BZ conversion was also high (almost 92%), while the overall Faradaic selectivities toward BA and HB formation were ∼48 and ∼44%, respectively. Lastly, it must be noted that the H_2_ formation rate on Cu/C in the presence of BZ (∼0.2 mmol_H_·g_Cu_^–1^·s^–1^; Figure 5a) was significantly lower than that in its absence (∼3.7 mmol_H_·g_Cu_^–1^·s^–1^; Figure 1a), suggesting low H* coverage in the presence of BZ.

**Figure 6:**
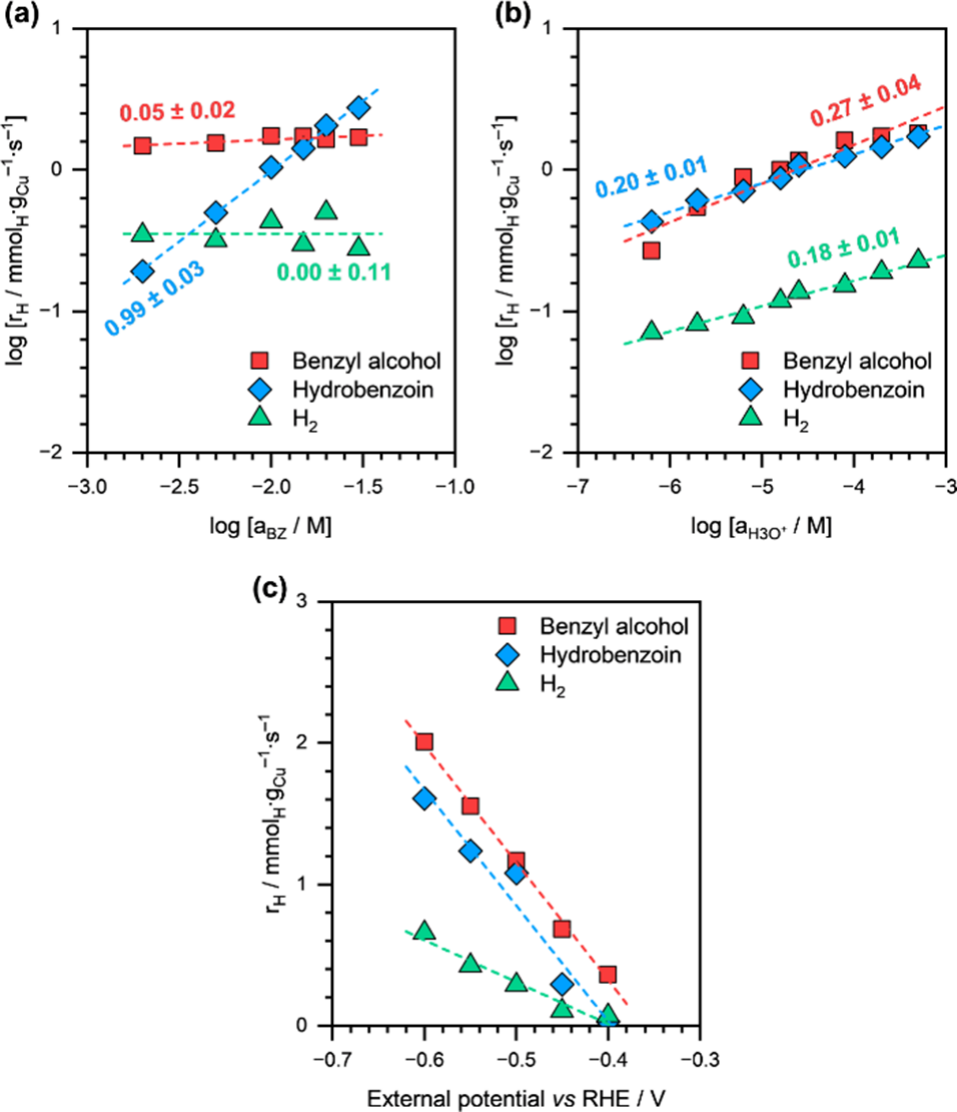
BA, HB, and H_2_ formation rates during BZ ECH on Cu/C, as a function of (a) initial BZ concentration (*a*_BZ_), (b) H_3_O^+^ activity (*a*_H_3_O^+^_), and (c) η. Reaction conditions: 2–30 mM BZ, η = −0.4 to −0.6 V vs RHE, 1.5 M acetate buffer solution (pH 3.2–6.3), room temperature, ambient pressure. The dashed lines are linear fits, and the reported numbers are estimated reaction orders.

Figure 6 shows the effects of initial BZ concentration (*a*_BZ_), *a*_H_3_O^+^_, and η during BZ ECH on Cu/C. The reaction orders in BZ for BA and HB formation were estimated to be ∼0.05 (approximately zero-order) and ∼0.99 (approximately first-order), respectively. HER, in the presence of BZ, also showed almost zero-order dependence on *a*_BZ_. We further note that the product formation rates increased modestly with *a*_H_3_O^+^_, and the reaction orders in H_3_O^+^ for BA, HB, and H_2_ formation were estimated to be ∼0.27, ∼0.20, and ∼0.18, respectively. Finally, it can be clearly seen that both BA and HB formation rates showed a similar dependence on η and increased with increasing overpotential.

We note here that *a*_BZ_, *a*_H_3_O^+^_, and η, all had an impact on the product selectivity during BZ ECH on Cu/C (Supplementary Figure S8). The HB selectivity gradually increased, while BA selectivity gradually decreased with increasing *a*_BZ_ (Supplementary Figure S8a). Similarly, HB selectivity initially increased with η (from −0.4 to −0.5 V vs RHE) but then remained invariant with a further increase in η (Supplementary Figure S8b). Lastly, electrolyte pH (or *a*_H_3_O^+^_) had no effect on BA and HB selectivity (Supplementary Figure S8c), and the two remained almost constant in the investigated pH range (3.2–6.3). Interestingly, the H_2_ Faradaic selectivity remained low (<0.1) under the investigated reaction conditions, indicating high FE toward BZ conversion.

**Figure 7:**
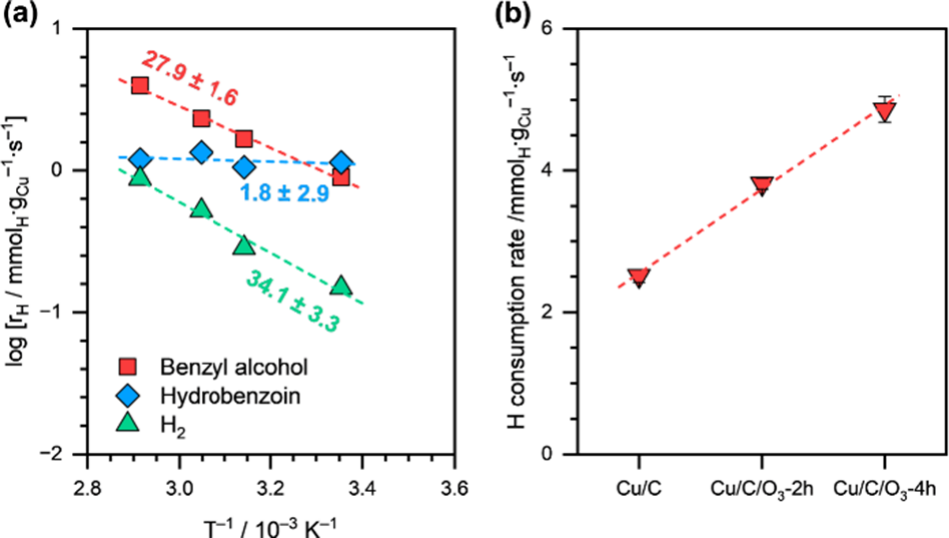
(a) Arrhenius-type plots for BA, HB, and H_2_ formation during BZ ECH on Cu/C. The dashed lines are linear fits, and the reported numbers are *E*_a_ in kJ·mol^–1^. (b) Total H consumption rates during BZ ECH on Cu/C, Cu/C/O_3_-2 h, and Cu/C/O_3_-4 h. Dashed line is a guide to the eye. Reaction conditions: 20 mM BZ, η = −0.5 V vs RHE, 1.5 M acetate buffer solution (pH ∼ 4.6), 298–353 K reaction temperature, ambient pressure.

Figure 7a shows the Arrhenius-type plots for BA, HB, and H_2_ formation during BZ ECH on Cu/C. The apparent activation energies (*E*_a_) for BA and HB formation were estimated to be ∼28 and ∼2 kJ·mol^–1^, respectively. The corresponding apparent pre-exponential factors were estimated to be approximately 7 × 10^4^ and 2 × 10^1^ mmol_H_·g_Cu_^–1^·s^–1^, respectively. The *E*_a_ value for BA formation on Cu/C was similar to that reported on Pt/C and Rh/C (∼25 and ∼21 kJ·mol^–1^ at η = −0.7 V vs Ag/AgCl, respectively).^28^*E*_a_ for H_2_ formation was estimated to be ∼34 kJ·mol^–1^ (Figure 7a), while the corresponding apparent pre-exponential factor was 1 × 10^5^ mmol_H_·g_Cu_^–1^·s^–1^. Interestingly, Sharifi-Asl et al. also estimated similar activation energy for HER on Cu electrode (*E*_a_ ∼ 32 kJ mol^–1^).^43^

Figure 7b shows the effect of acid functionalization of the carbon support on the total H consumption rates on Cu/C during BZ ECH. The individual rates and Faradaic selectivities of BA, HB, and H_2_ formation on the O_3_-treated catalysts are compiled in Supplementary Table S2. It must be noted that all catalysts showed at least 90% Faradaic efficiency toward BZ conversion. Interestingly, both O_3_-treated catalysts, i.e., Cu/C/O_3_-2h and Cu/C/O_3_-4h, showed increasingly higher H consumption rates for BZ ECH. In fact, the ECH rate almost doubled from ∼2.4 mmol_H_·g_Cu_^–1^·s^–1^ on Cu/C to ∼4.9 mmol_H_·g_Cu_^–1^·s^–1^ on Cu/C/O_3_-4 h. Furthermore, BA and HB formation rates also increased individually upon O_3_ treatment (Supplementary Table S2). Lastly, we note that the HER rates (in the presence of BZ) also increased substantially from ∼0.24 mmol_H_·g_Cu_^–1^·s^–1^ on Cu/C to ∼0.51 mmol_H_·g_Cu_^–1^·s^–1^ on Cu/C/O_3_-4 h.

### H_2_ Evolution during BZ ECH on Cu

Let us first discuss H_2_ formation on Cu/C in the presence of BZ. First, we note that the HER rate in the presence of BZ (∼0.2 mmol_H_·g_Cu_^–1^·s^–1^; Figure 5a) was much lower than the HER rate in its absence (∼3.7 mmol_H_·g_Cu_^–1^·s^–1^; Figure 1a). We concluded above that the kinetically rate-determining step for HER on Cu/C is the Tafel step and that the HER on Cu/C follows the Volmer–Tafel(rds) mechanism. As *r*_Tafel_ is second order in θ_H_ (eq 3), the substantially lower HER rate in the presence of BZ suggests that θ_H_ ≪ 1 under BZ ECH reaction conditions. Additionally, the almost zero-order dependence of BA formation on *a*_BZ_ (Figure 6a) indicates the high surface coverage of the organic substrate. Assuming competitive adsorption between BZ* and H* for the same sites on the Cu surface, we postulate that H* coverage must be low in the presence of BZ. The low H* coverage is also evidenced by the high FE toward BZ conversion on Cu/C (>90%) under BZ ECH reaction conditions.

Remarkably, the HER rates, in the presence of BZ, increased with increasing *a*_H_3_O^+^_ (Figure 6b) and η (Figure 6c). This trend contrasts with that observed in the absence of BZ, where HER rates showed almost no dependence on *a*_H_3_O^+^_ (Figure 3a). Furthermore, in the presence of BZ, O_3_ treatment of the carbon support almost doubled the HER rate from ∼0.24 mmol_H_·g_Cu_^–1^·s^–1^ on Cu/C to ∼0.51 mmol_H_·g_Cu_^–1^·s^–1^ on Cu/C/O_3_-4h (Supplementary Table S2). This result is again in contrast to the results obtained in the absence of BZ, where the HER rates remained almost invariant after the O_3_ treatment of the carbon support (Figure 3b). We recall here that at low H* coverages (i.e., θ_H_ ≪ 1), θ_H_ is dependent on the reversible rate of the Volmer step, which in turn is dependent on both *a*_H_3_O^+^_ and η (eq 1). The clear dependence of HER rates on *a*_H_3_O^+^_ and η, therefore, suggests that θ_H_ ≪ 1 under BZ ECH reaction conditions.

We further note here that both *r*_Tafel_ and *r*_Heyrovsky_ are dependent on θ_H_ (eqs 3 and 2, respectively); however, the kinetic dependence of *r*_Tafel_ on θ_H_ is second order, while that of *r*_Heyrovsky_ is first order. Therefore, under BZ ECH reaction conditions, it is also possible that *r*_Tafel_ < *r*_Heyrovsky_ at low H* coverages. In other words, when θ_H_ ≪ 1, the H_2_ formation could occur via the Heyrovsky step. As the Heyrovsky step follows the PCET mechanism, the change in the mechanism of H_2_ evolution from Volmer–Tafel to Volmer–Heyrovsky could also explain the dependence of HER rates on *a*_H_3_O^+^_ and η. Although it is not possible to confirm this shift in the mechanism from our kinetic investigations, we can unequivocally conclude that the surface coverage of H* is low (i.e., θ_H_ ≪ 1) on Cu/C under BZ ECH reaction conditions.

### Mechanism for BA Formation during BZ ECH on Cu

**Scheme 4:**
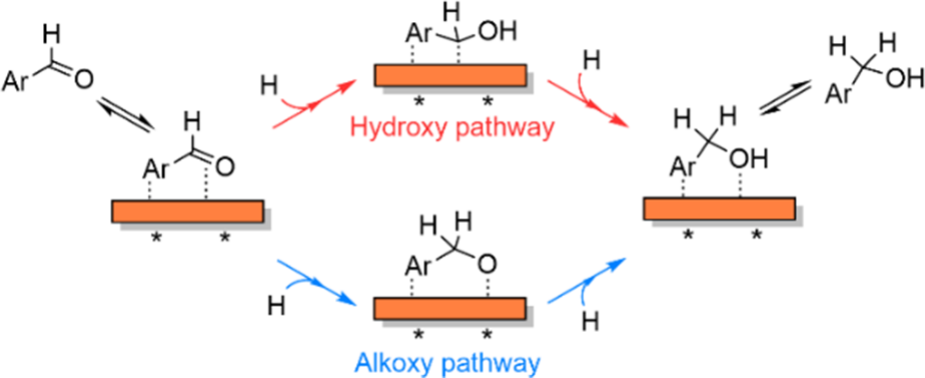
Mechanism for BA Formation during BZ ECH via the Alkoxy and Hydroxy Pathways

BA formation from BZ requires two successive H additions to the BZ molecule. These H additions have been postulated to proceed via two distinct pathways: the hydroxy pathway and the alkoxy pathway (illustrated in Scheme 4). In the hydroxy pathway, the carbonyl O of the adsorbed BZ (ArCHO*) is hydrogenated first to form a surface hydroxy intermediate (ArCHOH*), followed by a second H addition to its α-C to form BA. In the alkoxy pathway, on the other hand, the carbonyl C atom is first hydrogenated to form an alkoxy intermediate (ArCH_2_O*), followed by hydrogenation of O to form BA.

We performed isotope labeling studies to distinguish between the alkoxy and the hydroxy pathways for BA formation. Cheng et al. have previously shown that during BZ hydrogenation (on Pd/C), the addition of D to the carbonyl C via the alkoxy pathway forms ArCHDO* and would inevitably result in the formation of some deuterated BZ (ArCDO*) due to the reversibility of this step.^37^ On the other hand, addition of D to the carbonyl O via the hydroxy pathway forms ArCHOD*. The reverse reaction, in this case, would only form nondeuterated BZ (ArCHO*). Therefore, the presence of deuterated BZ in the electrolyte can be used to distinguish between the two pathways. In the case of BZ ECH, the solvent (i.e., H_2_O) is the source of H. Therefore, we performed BZ ECH on Cu/C using D_2_O as a solvent instead of H_2_O. However, we did not detect any deuterated BZ (i.e., ArCDO) in the electrolyte solution even after 90 min of reaction. These results, therefore, suggest that the hydroxy pathway must be the preferred pathway for H addition during BZ ECH on Cu.

We also note that HB formation during BZ ECH involves C–C coupling between two partially hydrogenated BZ. HB formation, therefore, necessitates H addition to the carbonyl O atom of each BZ and C–C bond formation between the corresponding α-C atoms. A preferential H addition to the carbonyl C via the alkoxy pathway would saturate the C atom and, therefore, inhibit C–C coupling. As HB was observed in significant quantities during BZ ECH (Figure 4a), we conclude that the hydroxy pathway is the preferred pathway for H addition on Cu/C under the investigated BZ ECH reaction conditions.

Let us now discuss the H addition mechanism during BZ ECH to BA. These H additions could occur either via the PCET mechanism or via the LH-type surface mechanism. The LH pathway involves the reaction of an adsorbed BZ with surface H* species. The PCET mechanism, on the other hand, involves an ER-type reaction of an adsorbed BZ with a solvated H_3_O^+^, coupled with a concerted electron transfer from the catalyst’s surface. The rate of H addition via the PCET mechanism (*r*_H,PCET_) can be expressed as
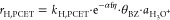
4where *k*_H,PCET_ is the kinetic rate constant and θ_BZ_ is the surface coverage of adsorbed BZ. The rate of H addition via the LH mechanism (*r*_H,LH_), on the other hand, takes the following form
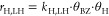
5where *k*_H,LH_ is the kinetic rate constant and θ_H_ is the surface coverage of H*. Based on these rate equations, we can clearly see that *r*_H,PCET_ depends on *a*_H_3_O^+^_ and η, while *r*_H,LH_ depends on θ_H_.

We recall that the BA formation rates showed a positive dependence on both *a*_H_3_O^+^_ (Figure 6b) and η (Figure 6c), and on O_3_ treatment of the carbon support (Supplementary Table S2), thus suggesting that the kinetically relevant step for BA formation (either the first or the second H addition) involves PCET. However, we must note here that these results do not exclude the H addition via the LH step. We have concluded above that θ_H_ ≪ 1 in the presence of BZ and that H* coverage increases with *a*_H_3_O^+^_ and η. The increase in θ_H_ would consequentially also increase *r*_H,LH_, thus explaining the above trends.

**Figure 8:**
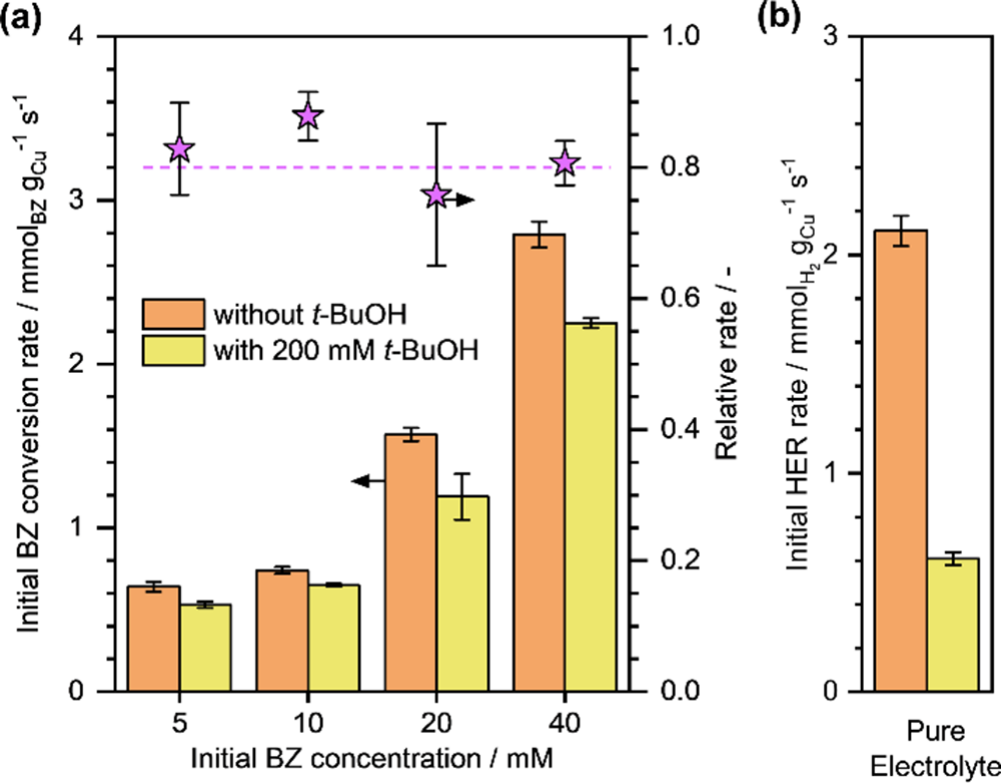
(a) Initial BZ conversion rates during BZ ECH and (b) initial HER rates in pure electrolyte solution on Cu/C with and without 200 mM *t*-BuOH. The relative rates in the presence of *t*-BuOH are also reported. Reaction conditions: 0–40 mM BZ, η = −0.5 V vs RHE, 1.5 M acetate buffer solution (pH ∼ 4.6), room temperature, ambient pressure.

To conclusively distinguish between PCET and LH pathways for H addition, we performed BZ ECH on Cu/C in the presence of *t*-butanol (*t*-BuOH), a specific quenching agent toward surface H*. The obtained results are presented in Supplementary Figure S9.^48−50^Figure 8a shows the initial BZ conversion rates (*r*_BZ_) on Cu/C at varying *a*_BZ_ in the presence or absence of *t*-BuOH. It can be clearly seen that *r*_BZ_ decreased in the presence of *t*-BuOH in all cases. This decrease clearly suggests that the LH pathway contributes, at least to some extent, toward H addition. Using *r*_BZ_ as a quantitative indicator, we estimate that the contribution of LH pathway toward H addition is ∼18%, irrespective of the initial BZ concentration. Based on these results, we conclude that PCET is the dominating pathway for H addition on Cu/C, contributing ∼82% toward H addition under the investigated reaction conditions.

We also performed HER on Cu/C (in pure electrolyte solution) in the presence of *t*-BuOH (Supplementary Figure S10), and the corresponding initial H_2_ formation rates are shown in Figure 8b. It can be seen that the HER rates, in pure electrolyte solution, decreased substantially from ∼2.1 mmol_H2_·g_Cu_^–1^·s^–1^ without *t*-BuOH to ∼0.6 mmol_H2_·g_Cu_^–1^·s^–1^ in the presence of ∼200 mM *t*-BuOH, i.e., a decrease of more than 70%. As we have noted above, the HER on Cu/C occurs via the Tafel step, which is an LH-type surface recombination of two H* species. A more significant decrease in the HER rates in the presence of *t*-BuOH, in this case, is therefore expected.

The protonation and electron-transfer processes during H addition have also been described as either inner-sphere or outer-sphere processes.^8,51^ The inner-sphere reactions occur on the electrode surface while outer-sphere reactions occur in the solvent layer and do not require a strong interaction between the reactants or intermediates and the electrode surface. Self-assembled monolayers of organothiols have been shown to inhibit the inner-sphere reactions but not the outer-sphere reactions.^8,52^ Therefore, we performed BZ ECH in the presence of 10 mM 2-mercaptobenzothiazole (MBT) to distinguish between the inner-sphere and outer-sphere reactions, and the results are reported in Supplementary Figure S11. It can be seen that both HB and BA formation decreased significantly in the presence of MBT, thus clearly indicating that the reactions involved in the formation of BA and HB during BZ ECH are inner-sphere processes and occur on the surface of Cu.

**Figure 9:**
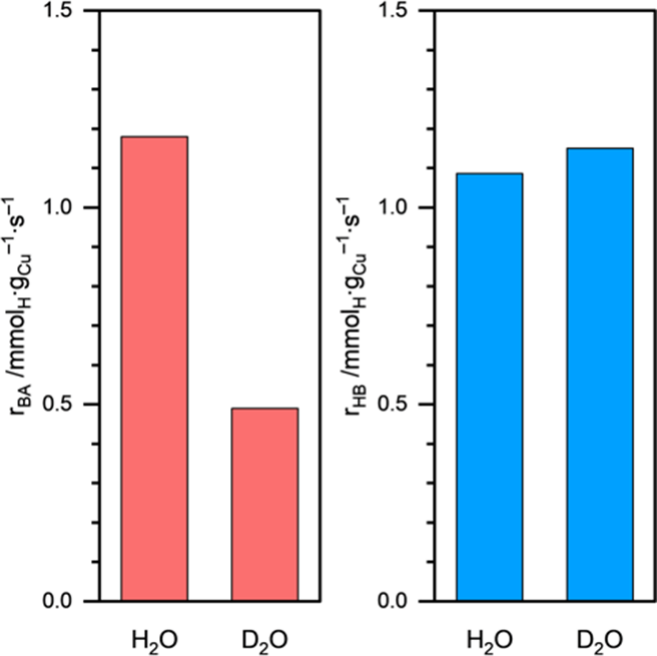
BA and HB formation rates during BZ ECH on Cu/C in H_2_O or D_2_O solvent. Reaction conditions: 20 mM BZ, η = −0.5 V vs RHE, 1.5 M acetate buffer solution (pH ∼ 4.6), room temperature, ambient pressure.

Next, to elucidate the kinetically relevant steps for BA and HB formation during BZ ECH, we performed isotope labeling experiments. Figure 9 shows the initial BA and HB formation rates during BZ ECH on Cu/C in H_2_O or D_2_O as the solvent. We can see that the BA formation rate decreased from ∼1.2 mmol_H_·g_Cu_^–1^·s^–1^ in H_2_O to ∼0.5 mmol_H_·g_Cu_^–1^·s^–1^ in D_2_O. This corresponds to a strong primary kinetic isotope effect (KIE) of *r*_H_/*r*_D_ = 2.4/1. The strong primary KIE suggests that H addition (either the first or the second) is the rate-determining step for BA formation. In contrast, the HB formation rate increased only slightly when the solvent was changed to D_2_O, corresponding to an inverse secondary KIE of *r*_H_/*r*_D_ = 1/1.08. The weak (secondary) KIE suggests that H is not directly involved in the kinetically relevant step for HB formation. Therefore, we conclude that the rate-determining step for HB formation is the C–C bond formation. The different rate-determining steps for BA and HB formation are also supported by the different *E*_a_ values (∼28 and ∼2 kJ·mol^–1^, respectively; Figure 7a).

### Mechanism for HB Formation during BZ ECH on Cu

**Scheme 5:**
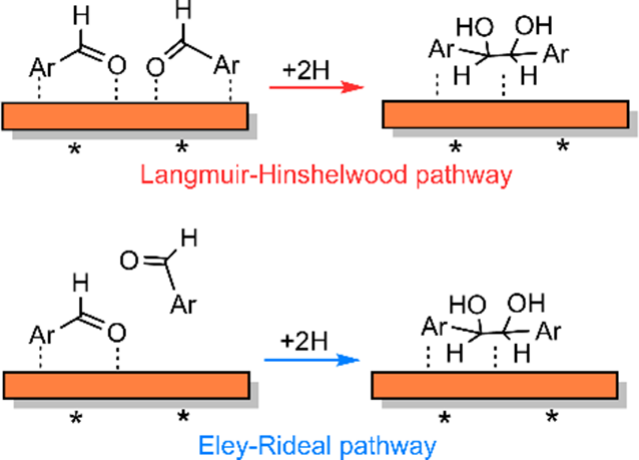
LH- and ER-type Pathways for C–C Coupling

Let us now focus on C–C coupling during BZ ECH on Cu/C. The C–C coupling during HB formation could occur either via an LH-type mechanism or via an ER-type mechanism (as illustrated in Scheme 5). The LH pathway involves a reaction between two surface intermediates, while the ER-type mechanism involves the reaction of an adsorbed intermediate with a physisorbed BZ molecule. The rates of C–C coupling via the LH-type (*r*_C–C,LH_) or the ER-type (*r*_C–C,ER_) mechanisms can be expressed as
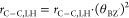
6
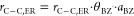
7where *k*_C–C,LH_ and *k*_C–C,ER_ are the respective kinetic rate constants, θ_BZ′_ is the surface coverage of the adsorbed intermediate, and *a*_BZ_ is the concentration of BZ.

We have shown above that, under the applied reaction conditions, the surface of Cu is saturated with the organic substrate, i.e., θ_BZ′_ ≈ 1. Therefore, under these conditions, the reaction orders in BZ for the LH-type and ER-type C–C coupling pathways are expected to be 0 and 1, respectively. We recall that the HB formation showed a reaction order of approximately 1 in *a*_BZ_ (Figure 6a). Therefore, we conclude here that HB formation on Cu/C proceeds via the ER-type mechanism wherein an adsorbed surface intermediate reacts with a physisorbed BZ.

HB formation, in addition to the C–C coupling step, requires H addition to the carbonyl O atoms of the involved BZ molecules. The proposed ER-type C–C coupling could, therefore, occur before or after the hydrogenation of the absorbed intermediate. In other words, the physisorbed BZ molecule could react with either (i) an adsorbed BZ* (ArCHO*), i.e., prior to the first H addition or (ii) a surface hydroxy intermediate (ArCHOH*) formed after the first H addition. It must be noted that the C–C coupling between a physisorbed BZ and adsorbed BZ* (ArCHO*) would form benzoin (following an intramolecular H transfer). However, we did not observe benzoin as a byproduct during BZ ECH on Cu/C. The possibility that any benzoin formed is rapidly hydrogenated to HB (under the applied reaction conditions) was investigated by performing ECH of benzoin on Cu/C under the same reaction conditions. The ECH of ∼20 mM aqueous solution of benzoin on Cu/C at η = −0.5 V versus RHE in 1.5 M acetate buffer solution (pH ∼ 4.6) showed negligible conversion to HB. These results, therefore, clearly suggest that the occurrence of C–C coupling before the first H addition step is unlikely. Therefore, we conclude that C–C coupling occurs after the first H addition and involves a partially hydrogenated surface hydroxy intermediate (ArCHOH*). The formation and stabilization of partially hydrogenated BZ (as ketyl radical species) on the surface of Cu has been observed experimentally.^19,20,33^

Finally, it is worth mentioning here that a fast second H addition to the surface hydroxy intermediate would result in the formation of primarily BA and, therefore, inhibit HB formation *via* C–C coupling. As HB was observed in significant quantities on Cu/C, we conclude that the second H addition has a lower rate constant than the first H addition and is, therefore, the rate-determining step for BA formation. The first H addition that forms the hydroxy intermediate can be assumed to be fast and equilibrated under the applied reaction conditions.

### Molecular Simulations

To further validate the postulated reaction pathways for BA and HB formation during BZ ECH on Cu, we simulated H addition and C–C coupling pathways using periodic DFT calculations on the Cu(111) surface (the most abundant facet evident from XRD). The simulations were performed on a system comprising a BZ molecule adsorbed flat (at ∼2.45 Å) on the Cu(111) surface. Charges were calculated using the Bader charge analysis. An implicit solvation model with additional explicit water molecules was employed in all simulations (refer to Experimental Section for more details on the computational methodology). Table 1 shows the calculated electronic energy barriers (Δ*E*_TS_) at 0 K for the first H addition, the second H addition, and the C–C coupling steps at the specified surface potentials relative to the RHE (*U*_*i*,RHE_). The *U*_*i*,RHE_ value of the Cu(111) surface was estimated from its work function (ϕ).

**Table 1:** Electronic Energy Barriers (Δ*E*_TS_) at 0 K of Different Reaction Steps for BA and HB Formation on Cu(111) Surface at the Specified Surface Potential (*U*_*i*,RHE_)

reaction step	Δ*E*_TS_/kJ·mol^–1^	*U*_*i*,RHE_^a^/V vs RHE
first H addition	26^b^, 46^c^	–0.42
second H addition	81^b^	–0.54
C–C coupling	36	–0.42

a Estimated at pH = 4.6 and *T* = 300 K.

b For PCET mechanism.

c For LH-type surface mechanism.

**Figure 10:**
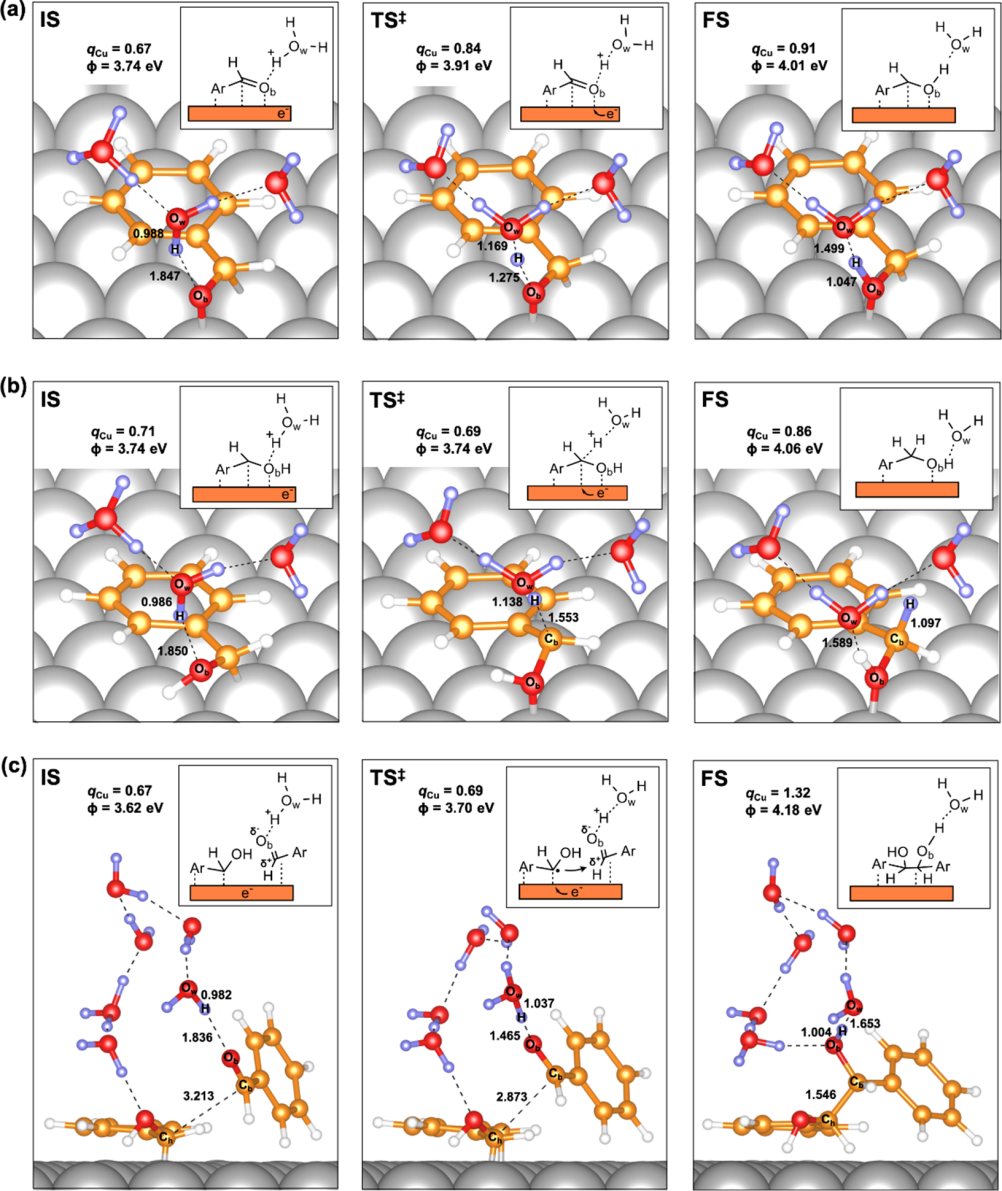
Initial (IS), transition (TS^‡^), and final states (FS) of (a) first H addition via PCET and (b) second H addition via PCET, for BA* formation, and (c) C–C coupling accompanied by PCET, for HB* formation on the Cu(111) surface. The estimated charges on the Cu surface (*q*_Cu_), determined using Bader charge analysis, and the corresponding work functions (ϕ) are also shown. The reported number are interatomic distances in Å. Cu: gray, C: orange, O: red, H: white/purple. Some H_2_O molecules have been removed for clarity.

Let us first look at the formation of surface hydroxy intermediate, i.e., first H addition, via the PCET mechanism. The first PCET step was simulated by adding a proton to the water layer above the adsorbed BZ molecule (and a corresponding electron to the surface). The initial (IS), transition (TS^‡^), and final states (FS) for the first PCET step are shown in Figure 10a. During the simulations, we observed that the carbonyl O of BZ (O_b_) was H-bonded to the nearby H_2_O_w_ (or H_3_O_w_^+^) molecules. In the PCET step, the proton was transferred from a H-bonded H_3_O_w_^+^ to O_b_, and the electron was simultaneously transferred from the Cu surface. An increase in the total charge on the Cu surface (from +0.67 to +0.91) indicated that the electron was transferred from the surface of the catalyst to the adsorbed intermediate. The energy barrier for the first PCET step was estimated to be ∼26 kJ·mol^–1^ (Table 1). First of all, the low-energy barrier suggests that the first H addition, in agreement with the experimental findings, is fast and can be assumed to be equilibrated under the applied reaction conditions. Additionally, we also noted that the total charge on the Cu surface and the O_b_–H interatomic distance in the TS^‡^ were closer to that in the FS compared to that in the IS, indicating a late transition state for the first H addition.

We also simulated the first H addition *via* the LH-type surface reaction between an adsorbed H* and an adsorbed BZ (illustrated in Supplementary Figure S12). The energy barrier for this step was estimated to be ∼46 kJ·mol^–1^ (Table 1). The relatively high barrier for the surface H addition corroborates our experimental findings that H addition on Cu/C occurs primarily *via* the PCET mechanism.

Next, we simulated the second H addition to the α-C of the surface hydroxy intermediate via PCET to form BA*. For this, we added another proton to the water layer above the adsorbed hydroxy intermediate (ArCHOH*) and a corresponding electron to the surface. We again observed that the hydroxy O (O_b_) was H-bonded to the nearby H_2_O_w_ (or H_3_O_w_^+^) molecules. In the second PCET step, H^+^ was transferred from a nearby H_3_O_w_^+^ to the α-C (C_b_) of the hydroxy intermediate, as illustrated in Figure 10b. The proton transfer was coupled with electron transfer from the surface of Cu. Similar to the first PCET step, the increase in the charge on the Cu surface (from +0.71 to +0.86) indicated that the electron was transferred from the surface to the adsorbed intermediate. The energy barrier for the second PCET was estimated to be ∼81 kJ·mol^–1^ (Table 1). We note that this barrier is significantly higher than that calculated for the first H addition (Δ*E*_TS_ ∼ 26 kJ·mol^–1^). In agreement with the experimental evidence, the higher energy barrier for the second H addition clearly suggests that it must be the rate-determining step for BA formation. The higher barrier for H addition to α-C (i.e., second H addition) compared to carbonyl O (i.e., first H addition) is likely due to the hydrophilic nature of the O atom. Lastly, the similar total charge on the Cu surface in the TS^‡^ and the IS, as well as a relatively large C_b_–H interatomic distance in the TS^‡^, indicates an early transition state for the second H addition.

Finally, let us discuss the formation of HB via the C–C coupling reaction between a surface hydroxy intermediate and a (physisorbed) BZ (illustrated in Figure 10c). For this, we placed a physisorbed BZ molecule next to the adsorbed hydroxy intermediate (as well as a proton to the water layer above and a corresponding electron to the Cu surface). The C–C bond formation involved an attack on the electrophilic carbonyl C (C_b_) of the physisorbed BZ molecule by the radical α-C (C_h_) of the hydroxy intermediate.

Interestingly, during the simulations, the C–C bond formation was accompanied by the protonation of the radical O (O_b_) of the formed alkoxy radical and a concerted electron transfer from the surface of Cu to form HB*. In other words, the C–C coupling was accompanied by PCET to directly form HB*. The increase in the charge on the Cu surface from the IS (+0.67) to the FS (+1.32) indicates electron transfer from the surface to the adsorbed intermediate. We also note that the total charge on the Cu surface in the TS^‡^ was similar to that in the IS, thus indicating that electron transfer had not occurred up to the transition state. The electronic energy barrier for the C–C bond formation step was estimated to be ∼36 kJ·mol^–1^ (Table 1).

Based on these first-principles molecular simulations, the electronic energy barriers (at 0 K) for BA and HB formation have been estimated to be ∼81 and ∼36 kJ·mol^–1^, respectively. These molecular simulations, therefore, suggest that the true activation energy barrier for BA formation must be higher than that for HB formation. This difference in the energy barriers agrees qualitatively with the experimentally observed apparent activation energies for BA and HB formation (∼28 and ∼2 kJ·mol^–1^, respectively; Figure 7a).

Finally, we note here that the mechanism of C–C coupling on Cu has been previously described in terms of the surface combination of two radical intermediates.^19,20^ The formation of these radical intermediates has been observed experimentally on Cu using infrared spectroscopy. Furthermore, the ability of Cu to stabilize these radical species has been proposed as a key descriptor of its ability to promote C–C coupling.^33^ However, based on the kinetic measurements (e.g., first-order reaction kinetics in *a*_BZ_ for HB formation), assisted by first-principles periodic DFT calculations, we propose here that the C–C coupling on Cu/C, at least under the investigated reaction conditions, follows an ER-type mechanism involving the reaction of a partially hydrogenated hydroxy (radical) intermediate with a physisorbed BZ molecule. The C–C bond formation is accompanied by a fast barrierless PCET to form HB.

### Overall Mechanism for BZ ECH on Cu

**Scheme 6:**
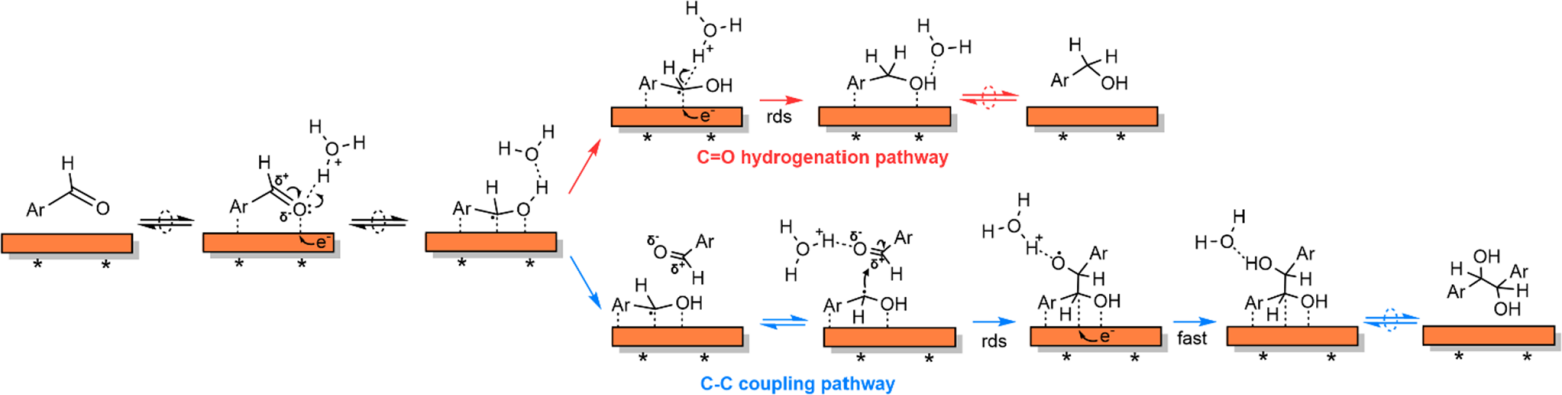
Proposed Mechanism for HB and BA Formation during BZ ECH on Cu/C

Combining the experimental and computational evidence, we now postulate the overall mechanism of BZ ECH to BA and HB on Cu/C (illustrated in Scheme 6). First, BZ (ArCHO) adsorbs reversibly on a vacant site (*) on the surface of Cu. The surface is predominantly covered with the organic substrate, and H* coverage is low under the applied reaction conditions. The adsorbed BZ (ArCHO*) undergoes a fast (and equilibrated) first PCET to the carbonyl O to form a surface hydroxy intermediate (ArCHOH*). The formed hydroxy intermediate follows two parallel reaction pathways. In the first pathway, ArCHOH* undergoes a second PCET on its α-C to form adsorbed BA*, which finally desorbs from the surface to form BA. This second H addition is the rate-determining step for BA formation. In a parallel reaction, the electrophilic carbonyl C of a nearby physisorbed BZ molecule (ArC^δ+^HO^δ-^) is attacked by the radical α-C of the hydroxy intermediate (ArC^•^HOH*) to form the C–C bond. The C–C coupling is accompanied by PCET to the radical O of the formed alkoxy radical intermediate to form HB*, which finally desorbs from the surface of Cu to form HB. The C–C bond formation is the rate-determining step for BZ ECH to HB.

## Conclusions

BZ ECH on Cu/C forms two primary products, viz., BA (the C=O hydrogenation product) and HB (the C–C coupling product), with an overall Faradaic efficiency of >90% at η = −0.5 V versus RHE. The Faradaic selectivities toward BA and HB formation were ∼48 and ∼44%, respectively. In the absence of an organic substrate, the H_2_ evolution on Cu/C follows the Volmer–Tafel mechanism, with the Tafel step being rate-determining. Under BZ ECH reaction conditions, the surface of Cu is predominantly covered with the organic substrate, and the coverage of H* is low. The adsorbed BZ undergoes a fast (equilibrated) first PCET on the carbonyl O to form a hydroxy intermediate. The hydroxy intermediate then undergoes a second (rate-determining) PCET on its α-C to form BA. In a parallel reaction, the electrophilic carbonyl C of a physisorbed BZ molecule is attacked by the radical α-C of the hydroxy intermediate to form the C–C bond. The C–C coupling is accompanied by a second PCET to form HB. The C–C coupling is the rate-determining step for HB formation.

## Experimental Section

### General Considerations

Benzaldehyde (99.5%), benzyl alcohol (99.8%), hydrobenzoin (99.0%), copper(II) acetate (99.9%), palladium(II) acetate (99.9%), acetic acid (99.8%), sodium acetate (99.0%), 2-propanol (99.5%), acetone (99.9%), diphenyl ether (99.0%), ethyl acetate (99.5%), *t*-butanol (99.5%), 2-mercaptobenzothiazole (97%), and D_2_O (99.9 atom % D) were purchased from Sigma-Aldrich and used without further purification. Deionized (DI) water (18.2 MΩ·cm^–1^) was used to prepare all aqueous solutions.

### Catalyst Synthesis

Cu/C (∼5 wt % metal loading) was prepared via the incipient-wetness impregnation method. An aqueous solution of copper(II) acetate was added dropwise to a Vulcan XC72R carbon black support (QuinTech), mixed thoroughly, and dried overnight at 333 K. The resulting dried material was first treated in 100 mL·min^–1^ N_2_ at 673 K (temperature ramp: 10 K·min^–1^) for 4 h, followed by reduction in 100 mL·min^–1^ H_2_ at 623 K (temperature ramp: 10 K·min^–1^) for 4 h. These reduced catalysts are referred to as Cu/C. For the preparation of ozone-treated Cu/C catalyst, the carbon black support was first treated in O_3_ at room temperature for 2–4 h, followed by the deposition of Cu via the incipient-wetness impregnation method described above. These O_3_-treated catalysts are referred to as Cu/C/O_3_-2h and Cu/C/O_3_-4 h, respectively.

The Pd/C (∼5 wt % metal loading) catalyst was also prepared via the incipient-wetness impregnation method described above using palladium(II) acetate as the metal precursor. The prepared Pd/C catalyst was first treated in 100 mL·min^–1^ N_2_ at 453 K (temperature ramp: 5 K·min^–1^) for 2 h, followed by reduction in 100 mL·min^–1^ H_2_ at 523 K (temperature ramp: 5 K·min^–1^) for 2 h.

### Carbon Felt Pretreatment

Carbon felt (Sigma-Aldrich, 3.0 cm × 1.5 cm) was immersed sequentially in acetone, DI water, and acetone again for at least 30 min each under ultrasonication treatment at ambient temperature. Finally, the treated carbon felt was dried in an oven at 333 K for 12 h.

### Electrode Preparation

The synthesized Cu/C (∼10 mg) was dispersed in a 1:1 solution of 1 mL 2-propanol and 1 mL DI water for 30 min under ultrasonication treatment at room temperature. The catalyst ink was then deposited on both sides of the pretreated carbon felt. The carbon felt electrode (containing Cu/C catalyst) was finally dried overnight in an oven at 333 K.

### Nafion Membrane Pretreatment

The Nafion-117 proton exchange membrane (Ion Power) was first immersed in a 3% H_2_O_2_ solution for 1 h at 353 K. The Nafion membrane was then immersed in DI water at 353 K for 2 h. Subsequently, the membrane was immersed in 1 mol·L^–1^ H_2_SO_4_ at 353 K for 1 h. Finally, the Nafion membrane was washed several times with DI water and stored in DI water under ambient conditions.

### Electrochemical Measurements

All electrochemical experiments were performed using a BioLogic VSP-300 workstation using a two-compartment batch electrolysis cell (Supplementary Figure S13). Cu/C deposited on a carbon felt was used as the working electrode. A double-junction Ag/AgCl (eDAQ) and a Pt wire (Sigma-Aldrich) were used as the reference electrode and counter electrode, respectively. The anode and cathode compartments were separated by the pretreated Nafion membrane. The reference Ag/AgCl electrode was calibrated against a reversible hydrogen electrode (RHE), and all the potentials herein are reported relative to the RHE, per the following equation.
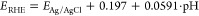
8where *E*_RHE_ and *E*_Ag/AgCl_ are electrode potentials relative to RHE and Ag/AgCl electrode potentials, respectively.

All electrochemical experiments were performed under ambient conditions. N_2_ gas (∼25 mL·min^–1^) was continuously bubbled through the electrolyte solution throughout the reaction to remove any dissolved gases. A stirring rate of 500 rpm allowed the complete dissolution of organics in the electrolyte solution and overcame the mass-transfer limitations (Supplementary Figure S14). The solution resistance between the working and reference electrodes was measured by potentiostatic electrochemical impedance spectroscopy (PEIS) and compensated (∼85%) by the electrochemical workstation. Prior to the reaction, the catalyst was polarized at −40 mA for ∼20 min to ensure the complete reduction of the metal.

The potentiodynamic linear sweep voltammetry (LSV) measurements were performed in either pure electrolyte solution or 20 mM BZ solution at a scan rate of 1 mV·s^–1^.

### Product Analysis

The course of the ECH experiments was followed by periodically withdrawing aliquots of ∼1 mL from the cathode compartment. The products in the aqueous electrolyte solution were extracted with ∼0.5 mL ethyl acetate. The extract was then analyzed by an offline gas chromatograph coupled with a mass spectrometer (Agilent 7890B GC/5977A MSD). The carbon balance between the reactants converted and formed products was >95% in all cases.

The initial rate toward a product *i* (in terms of H consumed per gram metal) was calculated from the product *i* formed versus reaction time plots using the following equation.
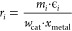
9where *m*_*i*_ is the initial slope, *w*_cat_ is the amount of catalyst, *x*_metal_ is the metal loading on the catalyst (as weight fraction), and ϵ_*i*_ is the number of H atoms required to form the product *i*. The initial slope corresponds to the linear region of the product *i* formed versus reaction time plot (typically 0–20 min).

The electron consumption toward H_2_ formation was indirectly estimated from the difference in the total electron consumption and the H (and the corresponding electron) consumption toward the formation of other products. The amount of H_2_ formed (*n*_H_2__) in mol was then calculated using the following equation
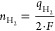
10where *q*_H_2__ is the electron consumption toward H_2_ formation, and *F* is Faraday’s constant. An exemplary plot is shown in Supplementary Figure S15. Additional calculation details are described in Supporting Information Section S3.

### Computational Methods

All quantum chemical calculations were performed on a periodic electrode–electrolyte interface model using the Vienna ab initio simulation package (VASP) with a plane-wave basis set.^53−56^ The simulations were performed on a 4 × 4 × 4 supercell of Cu(111) surface, cleaved from bulk Cu with a lattice constant of 3.59 Å, and a vacuum of at least 10 Å above the water layer. An implicit solvation model with additional explicit water molecules was used in all simulations. Detailed computations are provided in the Supporting Information Section S4.

## Supporting Information Available

Additional characterization details, supplementary data, additional calculation details, and additional computational details (PDF)
