# Supplementary material for: Mechanism of Electrocatalytic H2 Evolution, Carbonyl Hydrogenation, and Carbon–Carbon Coupling on Cu
Source: J Am Chem Soc. 2024 May 13;146(20):13949–61. doi: 10.1021/jacs.4c01911 (PMC11117180; doi:10.1021/jacs.4c01911)
Supplement: Supplementary file 1 — ja4c01911_si_001.pdf [file ja4c01911_si_001.pdf]

Supporting Information for

Mechanism of Electrocatalytic H_2 Evolution, Carbonyl Hydrogenation, and Carbon–Carbon Coupling on Cu

Hongwen Chen,[a] Jayendran Iyer,[b,c] Yue Liu,[d] Simon Krebs,[a] Fuli Deng,[a] Andreas Jentys,[a] Debra J. Searles,[c,e,f] M. Ali Haider,[a,b,g] Rachit Khare,[a]* and Johannes A. Lercher[a,h]*

[a] Department of Chemistry and Catalysis Research Center, Technical University of Munich, 85748 Garching, Germany

[b] Renewable Energy and Chemicals Laboratory, Department of Chemical Engineering, Indian Institute of Technology Delhi, 110016 New Delhi, India

[c] Australian Institute for Bioengineering and Nanotechnology, The University of Queensland, QLD 4072 Brisbane, Australia

[d] Shanghai Key Laboratory of Green Chemistry and Chemical Processes, School of Chemistry and Molecular Engineering, East China Normal University, 200062 Shanghai, China

[e] School of Chemistry and Molecular Biosciences, The University of Queensland, QLD 4072 Brisbane, Australia

[f] ARC Centre of Excellence for Green Electrochemical Transformation of Carbon Dioxide, The University of Queensland, QLD 4072 Brisbane, Australia

[g] Indian Institute of Technology Delhi–Abu Dhabi, Khalifa City B, Abu Dhabi, UAE

[h] Institute for Integrated Catalysis, Pacific Northwest National Laboratory, Richland, Washington 99352, United States

*Corresponding authors: rachit.khare@tum.de, johannes.lercher@ch.tum.de

Contents

S1. Additional Catalyst Characterization 3
S2. Supplementary Figures and Tables 7
S3. Additional Calculation Details 19
S4. Additional Computational Details 20
References 22

S1. Additional Catalyst Characterization

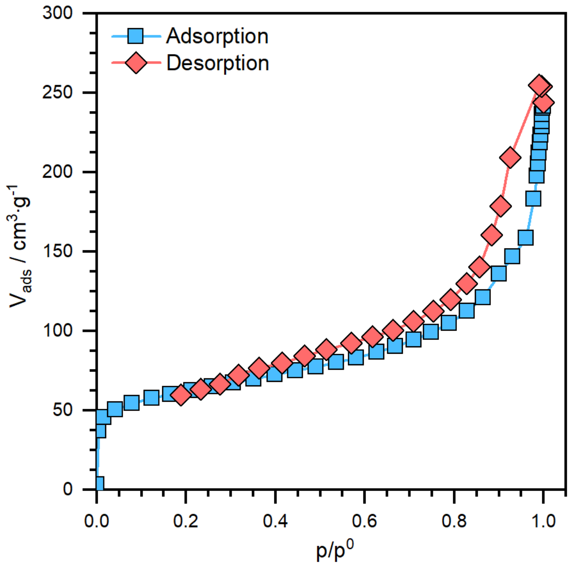

Figure S1. N_2 adsorption-desorption isotherm of Cu/C.

The N_2 adsorption-desorption isotherms were determined at 77 K on a PMI automated BET sorptometer. Prior to the measurements, the sample was outgassed at 523 K for 2 h. The surface area of the catalysts was estimated to be equal to ~223 $m^2 \cdot g_{cat}^{-1}$ by the Brunauer-Emmett-Teller (BET) method.

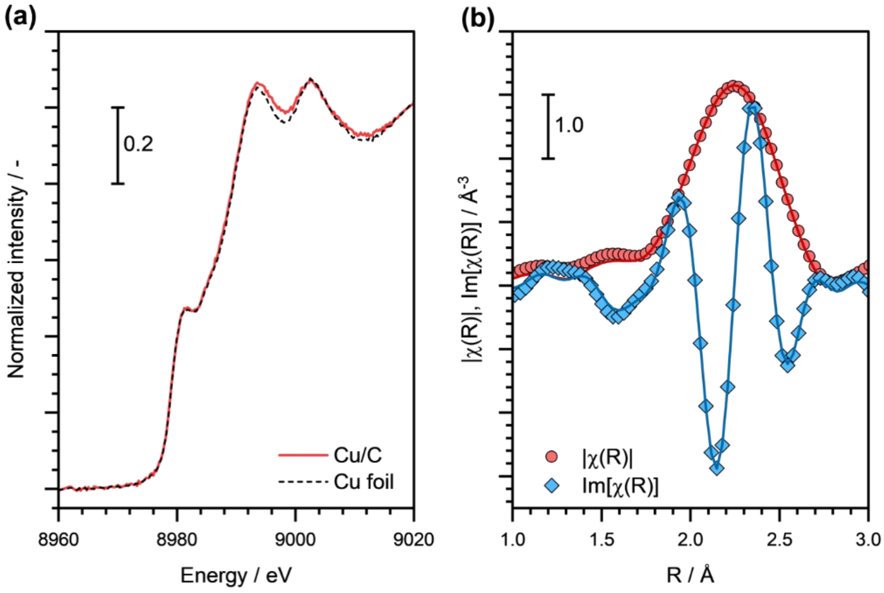

Figure S2. (a) X-ray absorption near edge structure (XANES) of Cu/C. The XANES of Cu reference foil is also shown for comparison. (b) Fourier-transformed extended X-ray absorption fine structure (FT-EXAFS) of Cu/C. Experimental data are shown as closed symbols and the corresponding fits are shown as solid lines.

Table S1. Fitting results: coordination numbers (CN), interatomic distances (d) and Debye-Waller factors (σ^2), for EXAFS of Cu/C and Cu reference foil. Fitting parameters: ${S_0}^2 = 0.93$, $\Delta E_0 = 4.5$ Å. The fitting was performed simultaneously on k^1-, k^2-, and k^3-weighted data in the q-space (k-range: 2.4 – 11.5 $Å^{-1}$ and R-range: 1 – 3 Å).

	CN_{Cu-Cu}	d_{Cu-Cu} /Å	σ^2 / $10^{-3} \times Å^2$
Cu/C	10.1 ± 0.6	2.545 ± 0.002	8.6 ± 0.5
Cu foil	12[a]	2.544 ± 0.002	9.0 ± 0.4

[a]This parameter was fixed during the fit.

The Cu K-edge X-ray absorption spectroscopy (XAS) measurements were performed on an easyXAFS300+ spectrometer system equipped with a ProtoXRD XRT60 X-ray tube and AXAS-M silicon drift detector (SDD) from KETEK GmbH. Monochromatic X-rays were obtained using a Si553 spherically bent crystal analyzer.

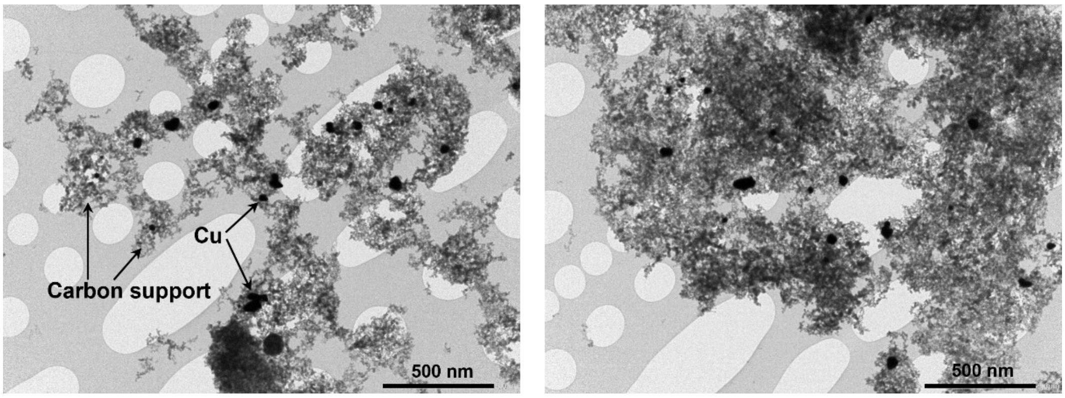

Figure S3. Transmission electron microscopy (TEM) images of Cu/C.

The TEM micrographs were recorded on a JEOL JEM-2011 scanning TEM with an accelerating voltage of 120 keV.

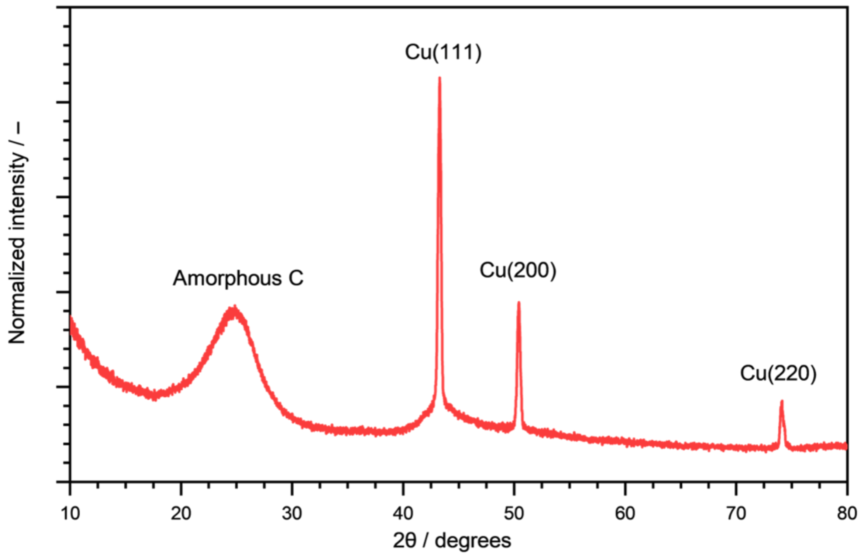

Figure S4. X-ray diffraction pattern of Cu/C.

The powder X-ray diffraction pattern was collected on a Philips X'Pert Pro System, with Cu-Kα radiation source operating at 45 kV and 40 mA. The sample was measured with a scanning rate of 0.02 deg·s^{-1} in 2θ range between 10° and 80°.

S2. Supplementary Figures and Tables

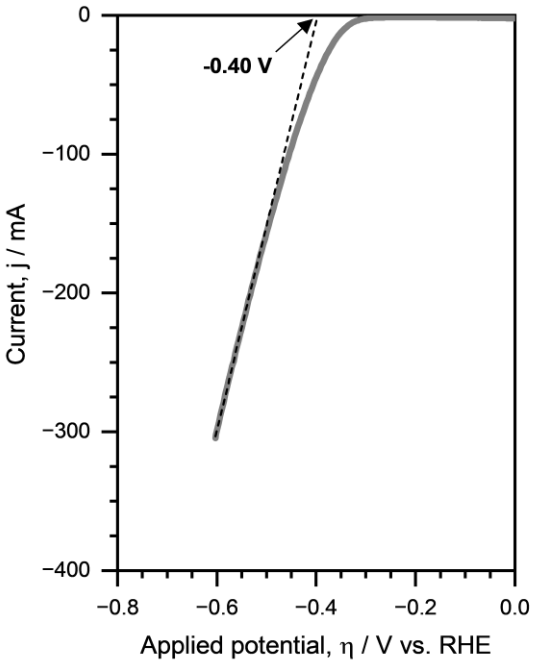

Figure S5. Linear sweep voltammetry (LSV) curve (scan rate = 1 mV·s^{-1}) on Cu/C in the presence of 20 mM benzaldehyde (BZ). Reaction conditions: 20 mM BZ, 1.5 M acetate buffer solution (pH ~ 4.6), room temperature, ambient pressure.

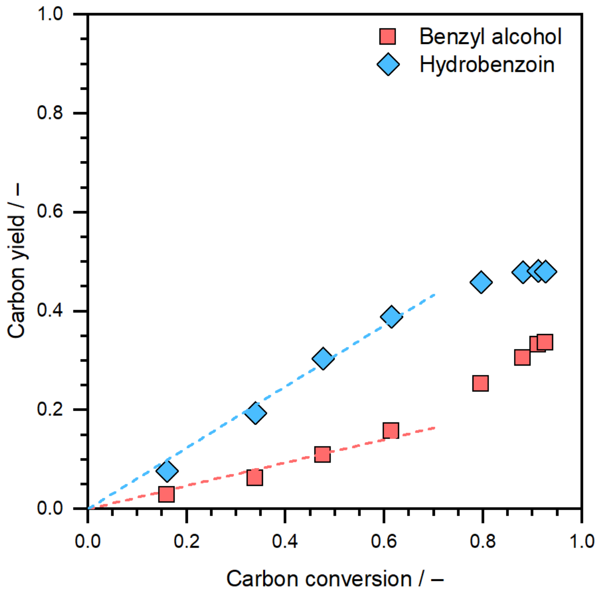

Figure S6. Yield versus conversion (on a carbon basis) plots during BZ ECH on Cu/C. Reaction conditions: 20 mM BZ, η = –0.5 V vs RHE, 1.5 M acetate buffer solution (pH ~ 4.6), room temperature, ambient pressure. The dashed lines are linear fits.

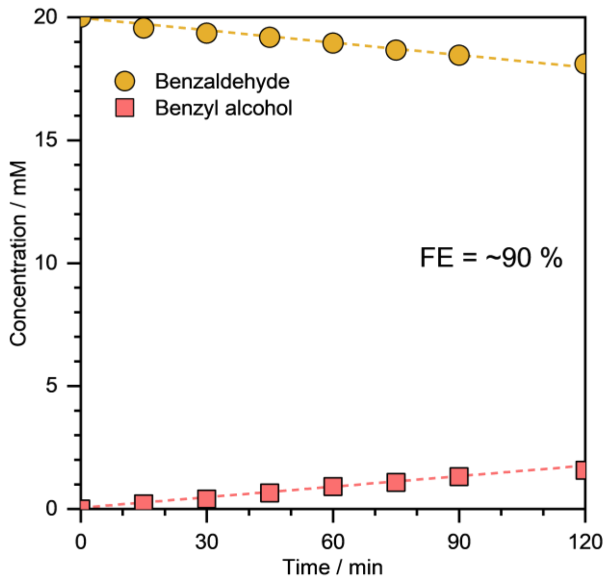

Figure S7. Concentration profiles of reactants and products during BZ ECH on the carbon black support. Reaction conditions: 20 mM BZ, η = –0.5 V vs RHE, 1.5 M acetate buffer solution (pH ~ 4.6), room temperature, ambient pressure. The dashed lines are guides to the eye. The reported number is the Faradaic efficiency (FE) towards BZ conversion.

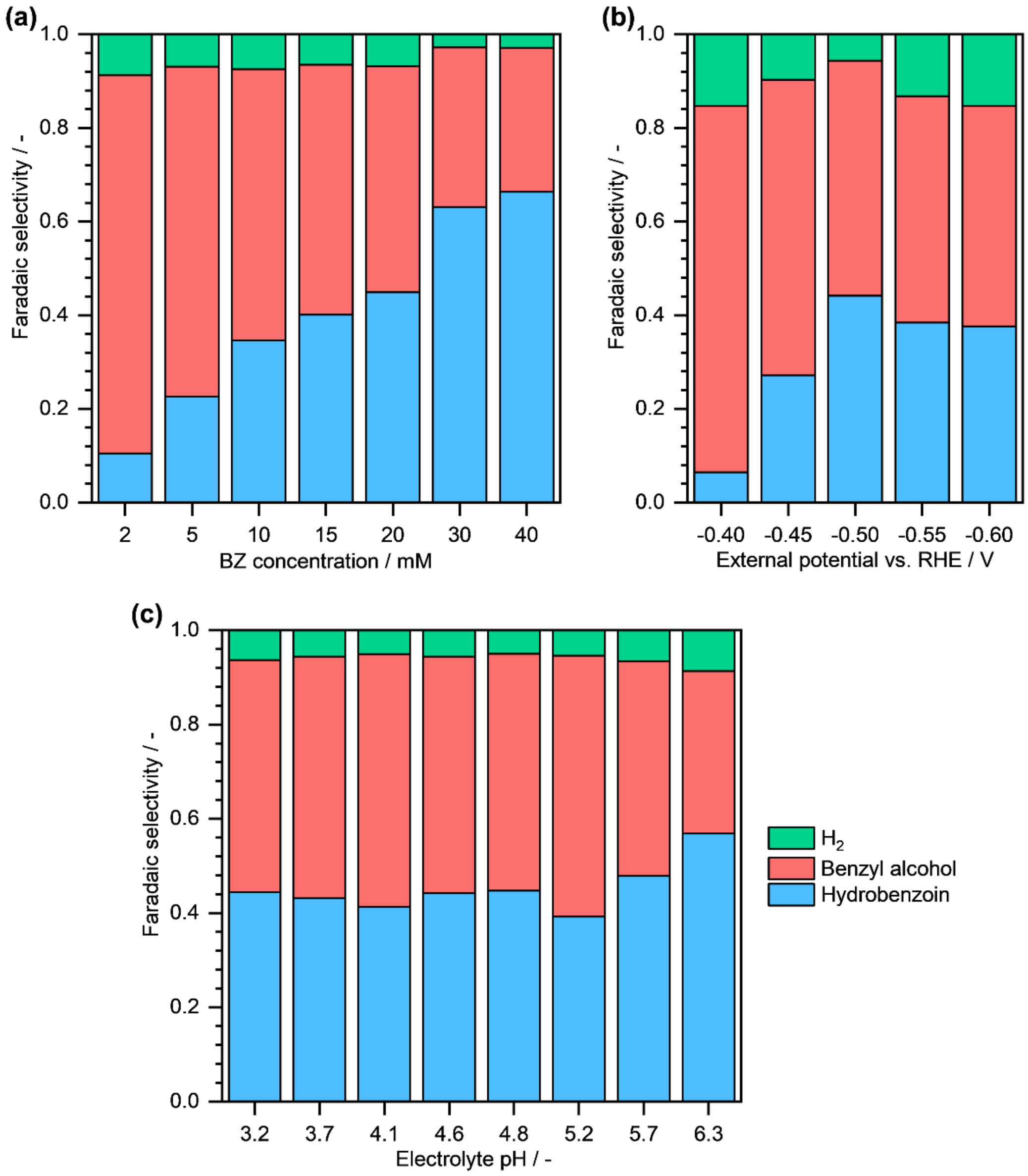

Figure S8. Faradic selectivities towards BA, HB, and H_2 during BZ ECH on Cu/C, as a function of (a) initial BZ concentration (a_{BZ}), (b) applied external potential (η), and (c) electrolyte pH. Reaction conditions: 2 – 40 mM BZ, η = –0.4 to –0.6 V vs RHE, 1.5 M acetate buffer solution (pH 3.2 – 6.3), room temperature, ambient pressure.

Table S2. Initial rates (in $mmol_{H}·g_{Cu}^{-1}·s^{-1}$) of BA, HB, and H_2 formation during BZ ECH on Cu/C, Cu/C/O_3-2h, and Cu/C/O_3-4h catalysts. The corresponding Faradaic selectivities are shown in parentheses. Reaction conditions: 20 mM BZ, η = –0.5 V vs RHE, 1.5 M acetate buffer solution (pH ~ 4.6), room temperature, ambient pressure.

	Cu/C	Cu/C/O_3-2h	Cu/C/O_3-4h
Benzyl alcohol	1.23 (0.48)	1.97 (0.48)	2.28 (0.44)
Hydrobenzoin	1.09 (0.43)	1.71 (0.42)	2.40 (0.46)
H_2	0.24 (0.09)	0.42 (0.10)	0.51 (0.10)

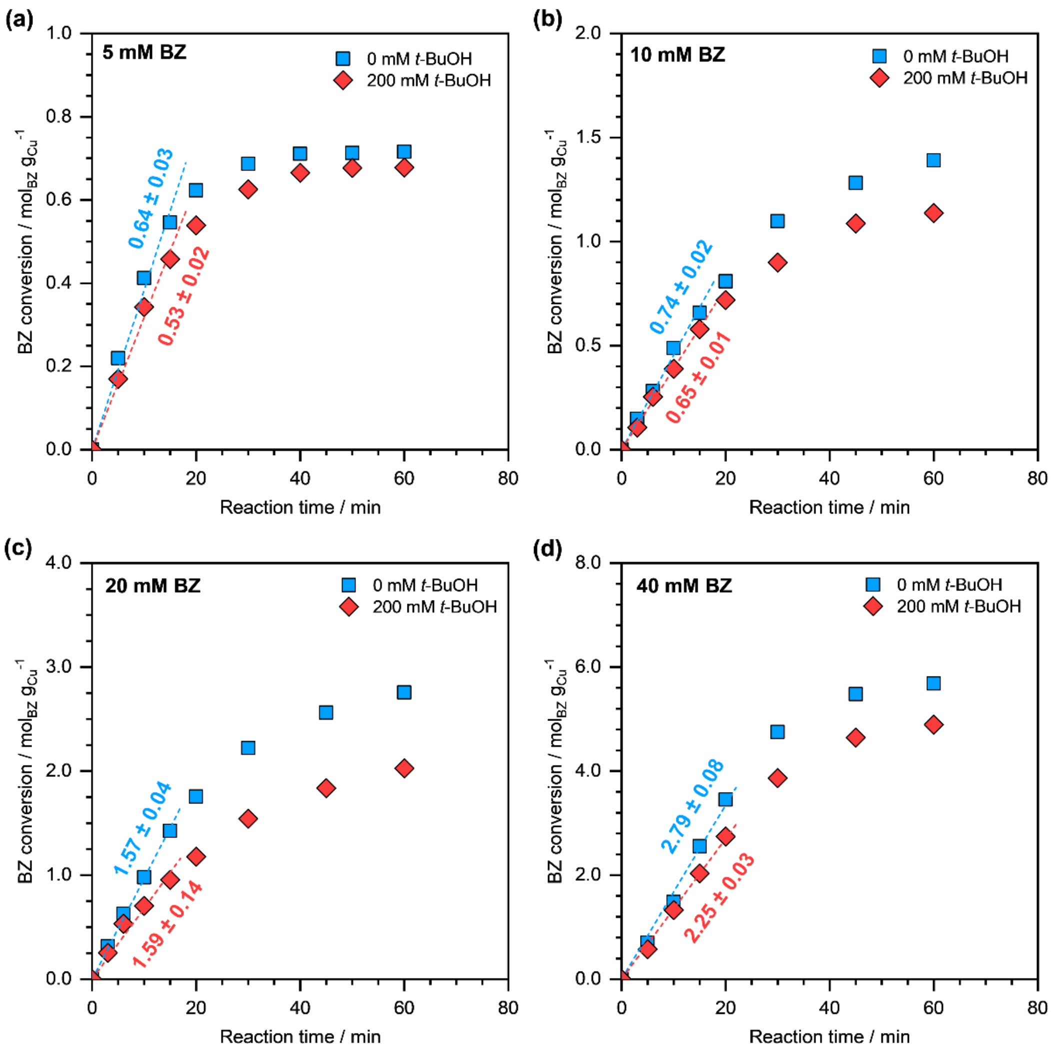

Figure S9. BZ conversion as a function of reaction time during BZ ECH with or without *t*-butanol (*t*-BuOH) on Cu/C with initial BZ concentration in the electrolyte equal to (a) 5 mM, (b) 10 mM, (c) 20 mM, and (d) 40 mM. Reaction conditions: 5 – 40 mM BZ, 0 mM or 200 mM *t*-BuOH, η = –0.5 V vs RHE, 1.5 M acetate buffer solution (pH ~ 4.6), room temperature, ambient pressure. The dashed lines are linear fits, and the reported numbers are the initial BZ conversion rates in $mmol_{BZ}\cdot g_{Cu}^{-1}\cdot s^{-1}$.

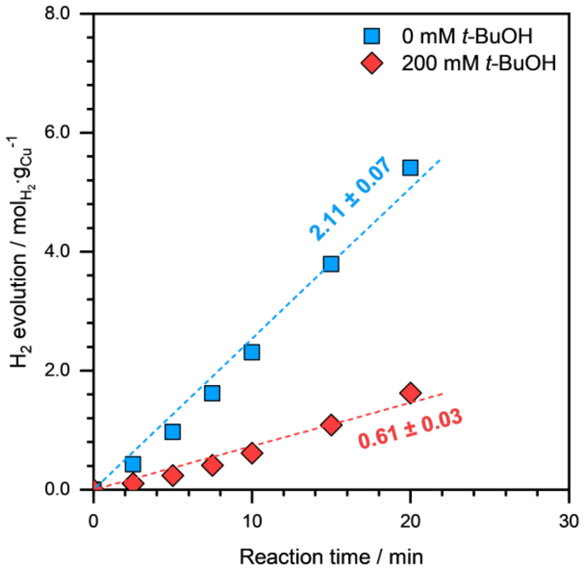

Figure S10. H_2 evolution as a function of reaction time during HER with or without *t*-BuOH on Cu/C. Reaction conditions: 0 mM or 200 mM *t*-BuOH, η = –0.5 V *vs* RHE, 1.5 M acetate buffer solution (pH ~ 4.6), room temperature, ambient pressure. The dashed lines are linear fits, and the reported numbers are the initial HER rates in $mmol_{H_2}\cdot g_{Cu}^{-1}\cdot s^{-1}$.

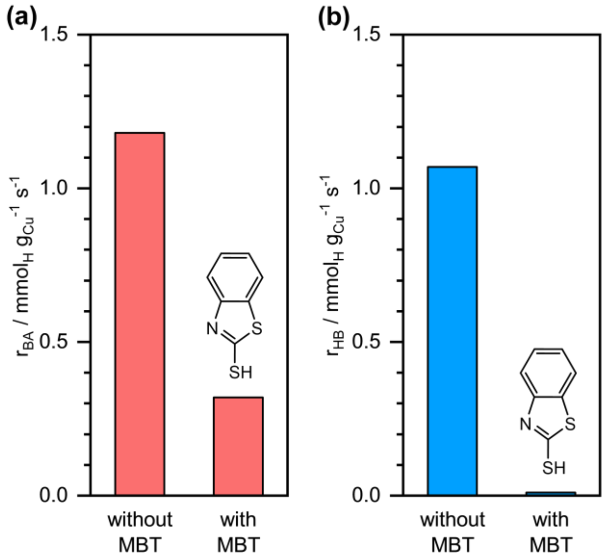

Figure S11. Initial BA (left panel) and HB (right panel) formation rates during BZ ECH on Cu/C with and without 10 mM 2-mercaptobenzothiazole (MBT). Reaction conditions: 20 mM BZ, 0 mM or 10 mM MBT, η = –0.5 V vs RHE, 1.5 M acetate buffer solution (pH ~ 4.6), room temperature, ambient pressure.

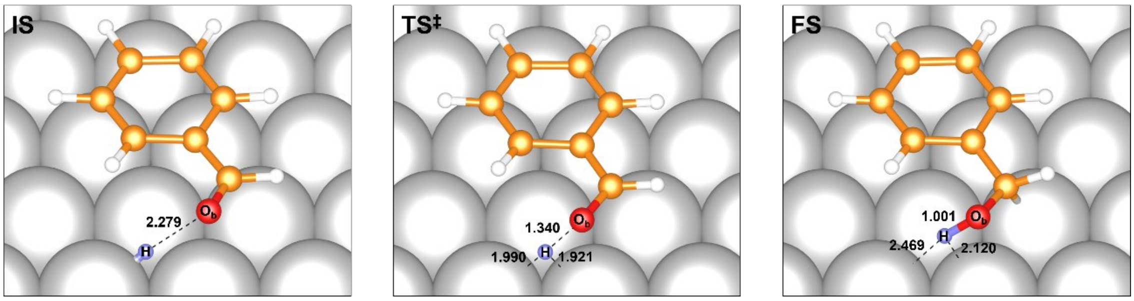

Figure S12. Initial (IS), transition (TS‡), and final (FS) states of first H addition to an adsorbed BZ molecule *via* the Langmuir-Hinshelwood-type surface reaction between BZ* and H* on the Cu(111) surface. The reported number are interatomic distances in Å. Cu: grey, C: orange, O: red, H: white/purple. H_2O molecules have been removed for clarity.

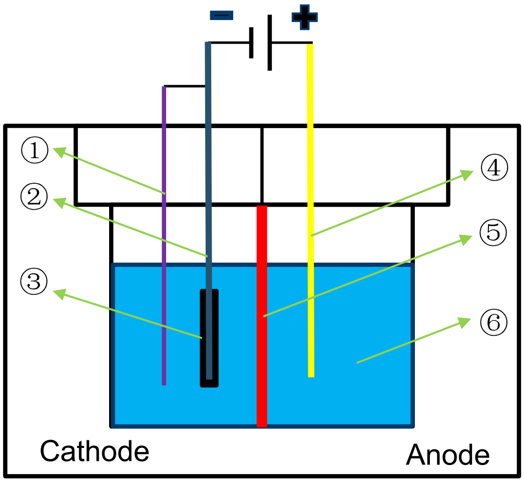

Figure S13. Schematics of the two-compartment electrochemical cell. (1) Ag/AgCl reference electrode. (2) Titanium rod. (3) Catalyst on a carbon felt (working electrode). (4) Pt wire (counter electrode). (5) Nafion proton-exchange membrane. (6) Electrolyte solution.

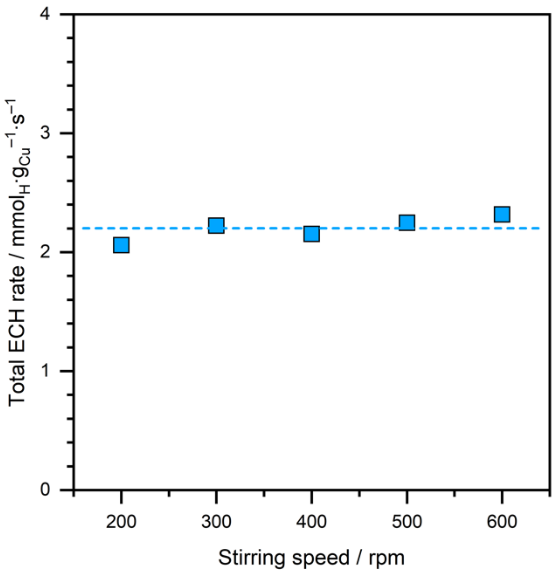

Figure S14. Total BZ ECH rate as a function of stirring speed of the rotor. Reaction conditions: 20 mM BZ, η = –0.5 V vs RHE, 1.5 M acetate buffer solution (pH ~ 4.6), room temperature, ambient pressure. The dashed line is a guide to the eye.

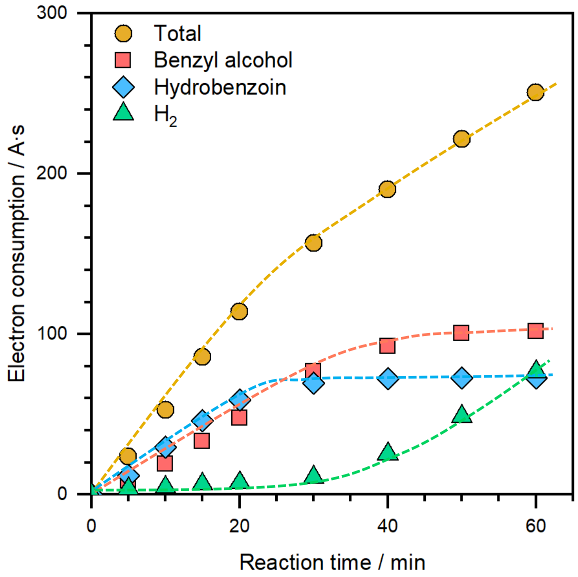

Figure S15. Electron consumption (in A·s) during BZ ECH on Cu/C. Reaction conditions: 20 mM BZ, η = –0.5 V vs RHE on Cu/C, 1.5 M acetate buffer solution (pH ~ 4.6), room temperature, ambient pressure. The dashed lines are guides to the eye.

The electron consumption (and the corresponding H^+ consumption) towards H_2 evolution (q_{H_2}) was calculated using the following relation.

$$q_{H_2} = q_{total} - q_{BA} - q_{HB}$$

where q_{total}, q_{BA}, and q_{HB} are the total electron consumption, and electron consumption towards BA and HB formation, respectively.

S3. Additional Calculation Details

The conversion of a reactant i at a given time t was estimated by:

$$\chi_i(t) = \frac{n_i(t)}{n_i^0}$$

where $n_i(t)$ is the amount of reactant detected at time t and n_i^0 is the initial amount of reactant in the electrolyte.

Similarly, the yield of a product j with respect to a reactant i at any given time t was estimated by:

$$Y_j(t) = \frac{n_j(t)}{n_i^0}$$

where $n_j(t)$ is the amount of product j detected at time t and n_i^0 is the initial amount of reactant i in the electrolyte solution.

The Faradaic efficiency towards organic conversion was estimated by:

$$FE_i = \frac{q_{org}}{q_{total}}$$

where q_{org} is the electrons consumed towards the conversion of the organic substrate and q_{total} is the total electrons consumed.

The Faradaic selectivity towards the formation of a product i was estimated by:

$$FE_i = \frac{q_i}{q_{total}}$$

where q_i is the electrons consumed towards formation of product i, and q_{total} is the total electrons consumed.

S4. Additional Computational Details

All quantum chemical calculations were performed on a periodic electrode-electrolyte interface model using the Vienna *ab initio* simulation package (VASP) with a plane wave basis set.[1-4] The single-electron wavefunctions were estimated using the employed basis set truncated at an energy cut-off of 400 eV. The k-points for these wavefunction calculations were sampled from a grid size 2 × 2 × 1 in the Brillouin zone. The partial occupancies were set for each orbital using the Methfessel-Paxton scheme to smoothen the wavefunctions.[5] Using projector augmented-wave (PAW) method, the core electrons were treated with the frozen-core approximations, while the valence electron wavefunctions were calculated using the plane wave basis set.[6] Further, to determine electron density from these wavefunctions, the electron self-interaction contribution in the Hartree potential was corrected using generalized gradient approximation (GGA) Perdew-Burke-Ernzerhof (PBE) exchange correlation functional with additional dispersion corrections including using the D3 method with the Becke-Johnson (BJ) damping scheme.[7, 8] The open-source VASPsol package was used to implement implicit solvent in all *ab initio* simulations with spin-polarized calculations.[9] The Bader charge analysis was performed using the code available from Henkelman and co-workers.[10]

All simulations were performed on a 4 × 4 × 4 supercell of the Cu(111) surface, cleaved from bulk Cu with lattice constant of 3.59 Å, and a vacuum of at least 10 Å above the water layer. The top two layers of the Cu(111) slab, representing the surface atoms were not constrained during simulations, while the bottom two layers were fixed representing bulk Cu. The electrode-electrolyte interface was modelled using 15 explicit H_2O molecules in addition to one proton and reaction intermediates above the Cu(111) surface. The system was first relaxed using *ab initio* molecular dynamics (AIMD) simulations. The AIMD simulations were performed using a canonical ensemble at constant temperature (300 K) and volume (NVT). To maintain constant temperature, a Nosé-Hoover thermostat was employed with a time constant of 0.01 ps. AIMD simulation were carried out for at least 5 ps with a time step of 0.5 fs while maintaining the energy and force convergence at 1×10^{-5} eV and 5×10^{-2} eV·Å^{-1}, respectively. The selected configurations from the AIMD simulations were further optimized using density functional theory (DFT). Three different reaction pathways were investigated: first H addition, second H addition, and C–C coupling. The H addition *via* PCET was modelled by including an additional H to the system that provided an H^+ to the water layer near the interface and an e^- to the metal surface, while the Langmuir-Hinshelwood type surface hydrogenation was modeled using H atoms (H*) adsorbed on Cu. The transition states (TS) for each step were identified using the climbing image-nudged elastic band technique (cl-NEB).[11] For this, 8 – 16 frames were developed between the initial state (IS) and the final state (FS) to observe a minimum energy path along the reaction coordinate.

We note that the work function (ϕ), or the surface potential (U), of the metal surface varies between the IS, TS, and FS, especially in the PCET steps. The obtained potentials were, therefore, corrected using the method developed by Chan and Nørskov.[12] Based on this method, the difference in the energy between state 1 and state 2 at a given potential can be estimated by:

$$E_2(\phi_1) - E_1(\phi_1) = E_2(\phi_2) - E_1(\phi_1) + \frac{(q_2 - q_1)(\phi_2 - \phi_1)}{2}$$

where ϕ_i is the work function at state i and q_i is the charge on the surface (including the adsorbate) at state i. For this, the charge was estimated using Bader charge analysis.

Furthermore, the work function (ϕ_i) at state i can be related to the surface potential of the Cu(111) surface using the following relation:

$$U_{i,SHE} = \frac{\phi_i - \phi_{SHE}}{e}$$

where $U_{i,SHE}$ is the surface potential of Cu(111) relative to SHE at state i, and ϕ_i and ϕ_{SHE} are the work functions of Cu(111) at state i and that of SHE (equal to 4.44 eV), respectively. Finally, the calculated surface potential is reported relative to the RHE ($U_{i,RHE}$), as per the following equation:

$$U_{i,RHE} = U_{i,SHE} + 0.0591 \cdot pH$$

References

(1) Kresse, G.; Furthmüller, J. Efficiency of ab-initio total energy calculations for metals and semiconductors using a plane-wave basis set. *Computational Materials Science* **1996**, *6* (1). DOI: https://doi.org/10.1016/0927-0256(96)00008-0

(2) Kresse, G.; Furthmüller, J. Efficient iterative schemes for ab initio total-energy calculations using a plane-wave basis set. *Physical Review B* **1996**, *54* (16). DOI: https://doi.org/10.1103/PhysRevB.54.11169

(3) Kresse, G.; Hafner, J. Ab initio molecular-dynamics simulation of the liquid-metal--amorphous-semiconductor transition in germanium. *Physical Review B* **1994**, *49* (20), 14251-14269. DOI: https://doi.org/10.1103/PhysRevB.49.14251

(4) Kresse, G.; Hafner, J. Ab initio molecular dynamics for liquid metals. *Physical Review B* **1993**, *47* (1). DOI: https://doi.org/10.1103/PhysRevB.47.558

(5) Methfessel, M.; Paxton, A. T. High-precision sampling for Brillouin-zone integration in metals. *Physical Review B* **1989**, *40* (6), 3616-3621. DOI: https://doi.org/10.1103/PhysRevB.40.3616

(6) Blöchl, P. E. Projector augmented-wave method. *Physical Review B* **1994**, *50* (24), 17953-17979. DOI: https://doi.org/10.1103/physrevb.50.17953

(7) Grimme, S.; Ehrlich, S.; Goerigk, L. Effect of the damping function in dispersion corrected density functional theory. *Journal of Computational Chemistry* **2011**, *32* (7), 1456-1465. DOI: https://doi.org/10.1002/jcc.21759

(8) Hammer, B.; Hansen, L. B.; Nørskov, J. K. Improved adsorption energetics within density-functional theory using revised Perdew-Burke-Ernzerhof functionals. *Physical Review B* **1999**, *59* (11), 7413-7421. DOI: https://doi.org/10.1103/physrevb.59.7413

(9) Mathew, K.; Sundararaman, R.; Letchworth-Weaver, K.; Arias, T. A.; Hennig, R. G. Implicit solvation model for density-functional study of nanocrystal surfaces and reaction pathways. *The Journal of Chemical Physics* **2014**, *140* (8), 084106. DOI: https://doi.org/10.1063/1.4865107

(10) Tang, W.; Sanville, E.; Henkelman, G. A grid-based Bader analysis algorithm without lattice bias. *Journal of Physics: Condensed Matter* **2009**, *21* (8), 084204. DOI: https://doi.org/10.1088/0953-8984/21/8/084204

(11) Henkelman, G.; Uberuaga, B. P.; Jónsson, H. A climbing image nudged elastic band method for finding saddle points and minimum energy paths. *The Journal of Chemical Physics* **2000**, *113* (22), 9901-9904. DOI: https://doi.org/10.1063/1.1329672

(12) Chan, K.; Nørskov, J. K. Electrochemical Barriers Made Simple. *The Journal of Physical Chemistry Letters* **2015**, *6* (14), 2663-2668. DOI: https://doi.org/10.1021/acs.jpclett.5b01043
